# The innate immune toll-like-receptor-2 modulates the depressogenic and anorexiolytic neuroinflammatory response in obstructive sleep apnoea

**DOI:** 10.1038/s41598-020-68299-2

**Published:** 2020-07-10

**Authors:** Dora Polsek, Diana Cash, Mattia Veronese, Katarina Ilic, Tobias C. Wood, Milan Milosevic, Svjetlana Kalanj-Bognar, Mary J. Morrell, Steve C. R. Williams, Srecko Gajovic, Guy D. Leschziner, Dinko Mitrecic, Ivana Rosenzweig

**Affiliations:** 1 Sleep and Brain Plasticity Centre, Department of Neuroimaging, Institute of Psychiatry, Psychology and Neuroscience (IoPPN), King’s College London (KCL), De Crespigny Park, Box 089, London, SE5 8AF UK; 2 University of Zagreb School of Medicine, Croatian Institute for Brain Research, Zagreb, Croatia; 3 BRAIN, Department of Neuroimaging, KCL, London, UK; 4 Department of Neuroimaging, IoPPN, KCL, London, UK; 5 School of Public Health, University of Zagreb School of Medicine, Zagreb, Croatia; 6 The National Heart and Lung Institute, Imperial College London, London, UK; 7 Department of Neurology, Guy’s and St Thomas’ Hospital (GSTT) and Clinical Neurosciences, KCL, London, UK; 8 Sleep Disorders Centre, GSTT, London, UK

**Keywords:** Neuroscience, Physiology, Medical research, Neurology, Pathogenesis

## Abstract

The increased awareness of obstructive sleep apnoea’s (OSA) links to Alzheimer’s disease and major psychiatric disorders has recently directed an intensified search for their potential shared mechanisms. We hypothesised that neuroinflammation and the microglial TLR2-system may act as a core process at the intersection of their pathophysiology. Moreover, we postulated that inflammatory-response might underlie development of key behavioural and neurostructural changes in OSA. Henceforth, we set out to investigate effects of 3 weeks’ exposure to chronic intermittent hypoxia in mice with or without functional TRL2 (TLR2^+/+^, C57BL/6-Tyrc-Brd-Tg(Tlr2-luc/gfp)Kri/Gaj;TLR2^−/−^,C57BL/6-Tlr2tm1Kir). By utilising multimodal imaging in this established model of OSA, a discernible neuroinflammatory response was demonstrated for the first time. The septal nuclei and forebrain were shown as the initial key seed-sites of the inflammatory cascade that led to wider structural changes in the associated neurocircuitry. Finally, the modulatory role for the functional TLR2-system was suggested in aetiology of depressive, anxious and anorexiolytic symptoms in OSA.

## Introduction

Obstructive sleep apnoea (OSA) is a major public health problem due to high prevalence and associated serious cardiovascular and metabolic complications^1,2^. Its neuropsychiatric presentations and links to anxiety disorders, depression^2,3^ and Alzheimer’s disease (AD) are also increasingly recognised^4–7^. The key mechanisms behind effects of OSA on the brain are unclear, and if established, they might aid the development of much needed novel therapeutic approaches.

More recently, neuroinflammation and microglial Toll-like receptors 2 (TLR2) system^8^ have been argued to act as a shared archetypal mechanism in the pathogenesis of AD, depression^9–11^ and several other psychiatric disorders^12–14^ with which OSA appears to share a complex bidirectional link^4,15^. Neuroinflammation, however, has yet to be authoritatively demonstrated in OSA.

Our group has long hypothesized that inflammatory response might arise during sleep in patients with OSA due to obstructive apnoeic events and associated intermittent hypoxia and arousals^16^. Over the years we have also argued that this neuroinflammatory process may drive specific structural and behavioural changes known to afflict some susceptible OSA patients^2,17^. In order to put this concept to test, we set out to investigate the effects of 3 weeks’ exposure to chronic intermittent hypoxia (IH) in an established mouse model of OSA^18,19^. This model has been shown to verifiably mimic the electroencephalographic arousals and significant hypoxaemia experienced by patients with OSA. In this study, its consequences were studied through time by multimodal in vivo and ex vivo approaches, combining magnetic resonance imaging (MRI) and bioluminescence imaging (BLI). The imaging was complemented by functional tests (weight monitoring, Y-maze, open-field and tail-suspension behavioural tests), imaging *versus* mRNA expression analysis, and ex vivo analyses of variety of cellular markers.

We report that TLR2 induction/microglial activation under OSA-like conditions initiates the inflammatory response in the brain’s forebrain and the septal nuclei, with a later widespread and marked chronic component. Moreover, we show that subsequent structural changes develop in distinct neuroanatomical regions with monosynaptic connections to initial frontal and basal forebrain cortical sites of inflammatory response. A significant modulatory role in the neuroinflammatory response is demonstrated for TLR2 and several other neuroplasticity molecules with known effects in brain ischemia^4,20,21^, including a cell adhesion molecule neuroplastin and the brain-derived neurotrophic factor (BDNF). Finally, we argue that neuroinflammation-driven changes in the distinct circuitry underlie several specific observed behaviours, including development of agitated (mal)adaptive behaviour under episodes of stress, and an increased ability to gain weight.

## Results

### *An acute TLR2 response* in the region of basal forebrain and the septal nuclei

**Figure 1:**
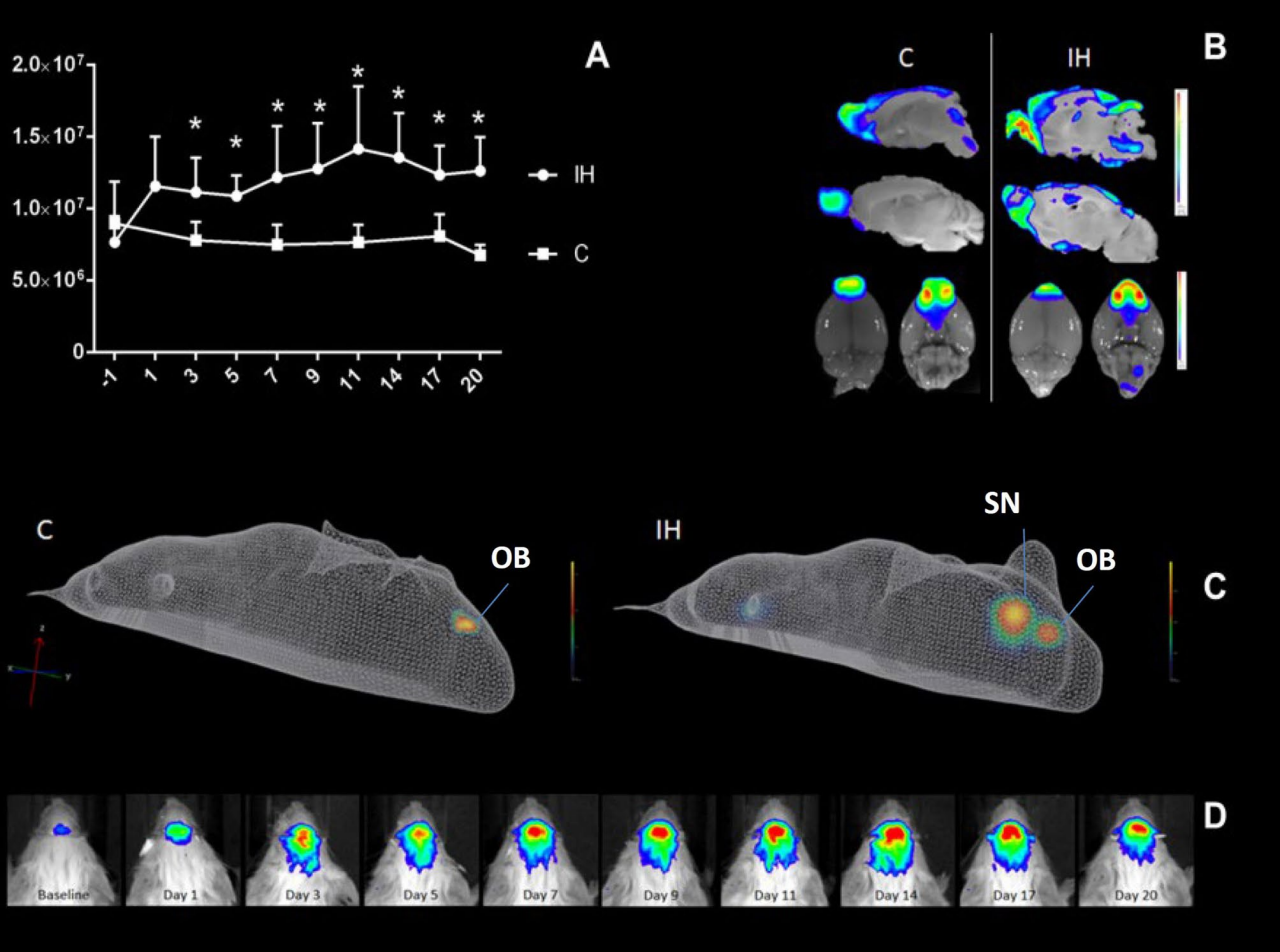
Neuroinflammatory response in a rodent model of obstructive sleep apnoea. In **A**, real-time in vivo bioluminescence imaging (BLI) of TLR2 response is shown. Plot of the data is depicted, which is obtained by measuring the luciferase activity (in photon per second, p/s) following intermittent hypoxia (IH; TLR2^+/+^IH; n = 7) and control (C; TLR2^+/+^CTRL; n = 6) conditions in TLR2^+/+^ mice. Statistically significant increase of neuroinflammatory response is recorded after 3 days of IH, which remained elevated throughout the protocol (mean ± SD; Wilcoxon signed ranks test, *P* < 0.05 *compared with baseline values). *X* *axis* shows BLI signal in photons/sec/cm2/sr, *Y* *axis* depicts day of IH protocol. In **B**, a representative image of ex vivo TLR2 signal is shown following 1 day of C (control) or IH (intermittent hypoxia) protocol in TLR2^+/+^ mouse. The signal is localized to the olfactory bulbs and anterior olfactory nucleus (OB) of both treatment groups at baseline. However, please note its stronger and more widespread posterior spread in IH group (TLR2^+/+^IH). In **C**, representative three-dimensional reconstruction images of diffuse light imaging tomography are shown following 3 days of IH and C protocol. A second locus of intense TLR2 expression is present in mice exposed to IH only (TLR2^+/+^IH), and it co-localises to the region of brain’s septal nuclei (SN). In **D**, photographs of TLR2 induction in the brain of a single TLR2^+/+^IH mouse are shown, taken at the same time each day, every day during the 3 weeks of investigations (e.g. day one, two three etc.). The colour calibrations at the right are photon counts. Note the occurrence of different scales in various ranges. *C* control, *TLR2* Toll like receptor 2, *IH* intermittent hypoxia, *SN* septal nuclei, *SD* standard deviation, *OB* olfactory bulb.

Previous studies have shown that TLR2 regulates the hypoxic/ischaemic brain damage caused by stroke^4,20,21^. To establish whether IH provokes a similar inflammatory response in brain, we used an established mouse model of OSA^19,22^ and transgenic mice that bore the dual reporter system luciferase/green fluorescent protein under transcriptional control of the murine TLR2-promoter. TLR2 induction/microglial activation and its spatial and temporal dynamics were investigated in real time using BLI^20^. As previously reported by us and others^20^, the luciferase immunoreactivity co-localized in more than 95% of cells with *Iba1* immunostaining^20^ (e.g. microglial marker; Supplement). The signals were analysed over a 3-week period following experimental (IH) and control (CTRL) protocol (Fig. 1A–D; also see SI Figure S1).

Using this relatively novel in vivo imaging approach, we demonstrated an inflammatory response with a marked chronic component. As shown in Fig. 1A, the photon emission and TLR2 signals/microglial activation were significantly elevated under IH conditions throughout a 3-week period interval. The quantitative analysis of photon emissions revealed that levels of TLR2-induction signals increased after 24 h (1.16 × 10^7^ ± 3.43 × 10^6^ p/s/cm^2^/sr, n = 18), it reached statistical significance after 72 h (Wilcoxon-test, Z = − 2.36, *P* = 0.004; baseline: 7.68 × 10^6^ ± 1.81 × 10^6^ p/s/cm^2^/sr, n = 20 vs day three: 1.12 × 10^7^ ± 2.40 × 10^6^, n = 17) (Fig. 1A) and thereafter it remained significantly elevated (Fig. 1A,D).

Olfactory bulb microglia in mice receive and translate numerous inputs from the brain and the environment and likely serve as sensors and/or modulators of brain inflammation. The subset of olfactory bulb microglial cells in mice was previously shown to continually express TLR2, enabling them to survey the environment in a ‘primed’ or alert state^20^. Similarly, in our study, a baseline activation over the area of the olfactory bulb and anterior olfactory nucleus was recorded (TLR2^+/+^CTRL: 7.655 × 10^6^ ps^−1^; n = 6; TLR2^+/+^IH: 7.821 × 10^6^ ps^−1^; n = 7; Fig. 1D; SI Fig. S8). Following 72 h of IH protocol, a significant activation in the region of the basal forebrain was further recorded only in TLR2^+/+^IH mice, which was traced to the region of the septal nuclei via the 3D in vivo reconstruction of the signal(Fig. 1C; SI Fig. S8).

### TLR2 modulates the effects of chronic IH on structural brain changes

**Figure 2:**
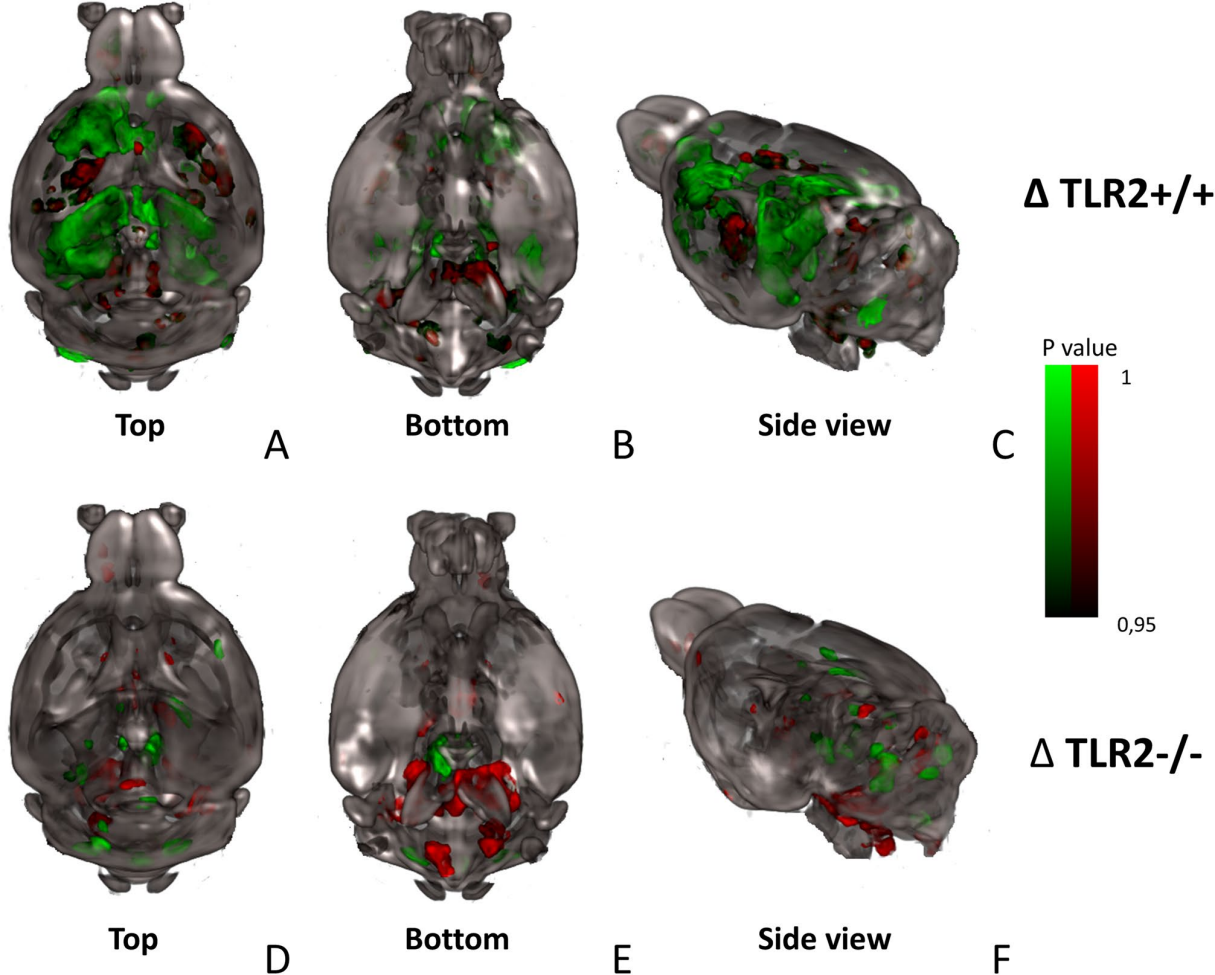
Three-dimensional rendering of all significant volume changes following 3 weeks of intermittent hypoxia protocol in mice with (TRL2^+/+^: top row) and without TLR2 receptors (TLR2^−/−^: bottom row). Co-existent hyper- (green clusters) and hypotrophic differences (red clusters) in the mouse brains are demonstrated. Shown contours are statistically significant differences, i.e. intermittent hypoxia (IH) > control (C); *P* < .01. Data is displayed on the mouse template image^63^^,^^62^. *3D* reconstructions are shown from 3 perspectives: (**A**,**D**) top view. (**B**,**E**) Bottom view. (**C**,**F**) Side view. *Hippocampus* is enlarged after IH as well as left part of the *motor* and *cingulate* *cortex* and *septum*, while these changes are not visible in TLR2^−/−^ mice. Suggested reductions of volume after IH are visible in the *reticular* *nuclei* of the *thalamus* and *dorsal* *striatum* of TRL2^+/+^IH and pronounced in *pons*, *medulla* and *mesencephalon* of TLR2^−/−^ IH. *3D* three-dimensional, *TLR2* Toll like receptor 2, *IH* intermittent hypoxia.

Neuroimaging of patients with OSA demonstrates distinct neuroanatomical changes^2^. In order to investigate whether the observed neuroinflammatory response under our experimental conditions leads to similar structural changes, we utilised high resolution ex vivo magnetic resonance imaging (MRI). To fully verify involvement of the TLR2-system, mice with (TLR2^+/+^) and without (TLR2^−/−^) functional TLR2 gene were imaged after 3 weeks of IH or CTRL protocol (Fig. 2; SI Figs. S2–S4).

**Figure 3:**
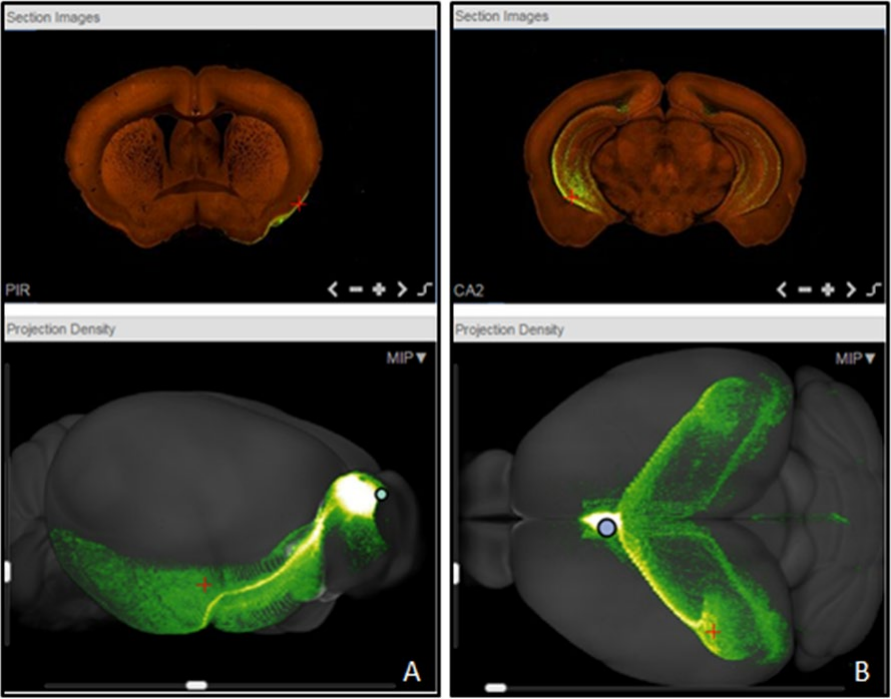
Representative coronal sections (first row) depict injection sites of rAAV tracer into the olfactory bulb (**A**), and septal nuclei (**B**) respectively (adapted from Allen Brain Atlas^64^). 3D tracing (second row) of distinct monosynaptic pathways from injected two regions is shown. (**A**) Monosynaptic pathways from OB are visualised; pathways can be visualised to traverse posteriorly and extend to the entorhinal cortex (right view). (**B**) Septal nuclei’ monosynaptic projections innervate wide bilateral hippocampi regions (top view)^64^. Of note is that the affected anatomical regions correspond to neuroanatomical structural changes observed following 3 weeks of our sleep apnoea model in mice with functional TLR2. *rAAV* retrograde labelling with adeno-associated virus, *TLR2* Toll like receptor 2.

As shown in Fig. 2, comparison of structural brain grey and white matter changes with MRI in TLR2^+/+^IH vs TLR2^+/+^CTRL mice demonstrated coexistent hyper- (*green*) and hypotrophic (*red*) cortical, subcortical and white matter changes. Most important enlargements were visible bilaterally in the hippocampi and presubiculi regions while the reduction of volume was most evident bilaterally in the reticular nuclei of the thalamus, dorsal striatum, parahippocampal and piriform cortex and dorsolateral pons, with involvement of distinct parts of periaqueductal grey (PAG), including the dorsal raphe nuclei (DRN) (Fig. 2, also see Supplement). Taken together, the spatio-temporal nature of the demonstrated neuroinflammatory process over the 3 weeks suggested that majority of later structural changes developed in neuroanatomical regions with monosynaptic connections (see Fig. 3) to initial frontal and basal forebrain cortical sites of microglial TLR2 response (Fig. 2).

Conversely, significantly weaker and predominantly hypotrophic changes were shown in TLR2^−/−^ mice (Fig. 2; SI Fig. S2). Here, changes after 3 weeks were located in more posterior and inferior brain regions of mesencephalon, pons and medulla, including important sleep regulating brainstem structures of locus coeruleus, parafacial, parabrachial, PAG, dorsal raphe and pedunculopontine nuclei (Figs. 2; SI Fig. S4).

In addition to structural changes observed by MRI, further immunochemistry analyses examined the effect of TLR2 genotype on the cellular level. The TLR2 genotype appeared protective against demyelinating effects of IH. For example, a widespread IH-induced demyelination of the hippocampus was only demonstrable in TLR2^−/−^ deficient mice (SI Fig. S7; F = 6.98, *P*_TLR2_^−/−^_IH_vs_TLR2_^−/−^_CTRL_ = 0.0286), whilst it did not reach statistical significance in mice with functional TLR2^+/+^. However, despite distinct morphological and cytoskeletal differences between astrocytes and microglia in four investigated groups, no statistically quantifiable changes in numbers of astroglia cells were recorded in respective regions of interest (ROIs) (SI Figs. S5, S6).

**Figure 4:**
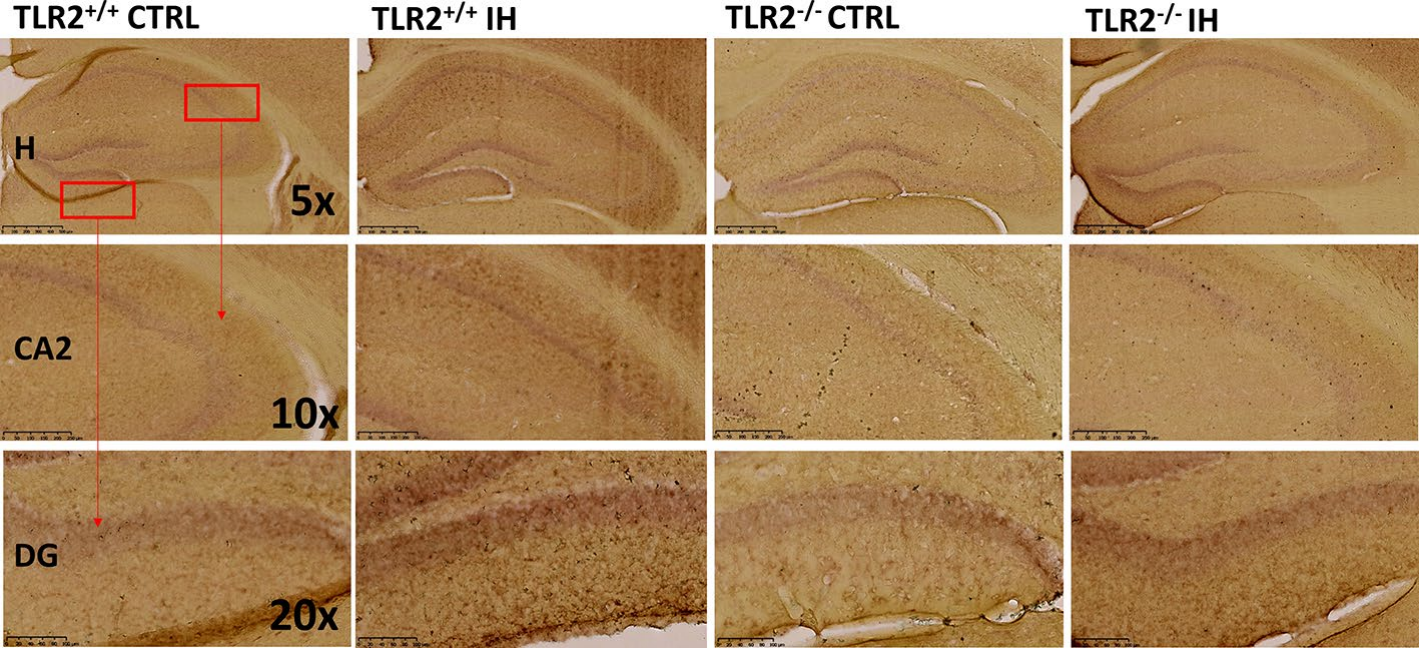
Representative images of neuroplastin staining in the hippocampus. Significantly lower neuroplastin immunoreactivity was recorded in TLR2 knock out mice (e.g. TLR2^−/−^CTRL and TLR2^−/−^IH) irrespective of the experimental protocol in several hippocampal (H) regions, including dentate gyrus (DG) and cornu ammonis 2 (CA2) layers. Scale bar in the first row (×5) where the whole hippocampus (H) is shown denotes 500 μm; the middle row (×10) shows CA2, and the bar denotes 250 μm; finally, in the bottom row (×20), DG layer is shown, here scale bar denotes 100 μm. *CA* Cornu Ammonis, *CTRL* control conditions, *DG* dentate gyrus, *H* hippocampus, *IH* intermittent hypoxia protocol, *TLR2* Toll like receptor 2.

Potential molecular drivers of noted enlargements in the ROIs in TLR2^+/+^IH and TLR^−/−^IH mice were then further explored (Fig. 4; SI Table S2). Of those, notably, we demonstrated a prominent up-regulation of the cell adhesion molecule neuroplastin in mice with a functional TLR2-system. This was shown by its strong immunoreactivity signal pattern in all major hippocampal sublayers that contain neurons [e.g. granular layer in dentate gyrus (DG), and pyramidal layers in *Cornu* *Ammonis* (CA)] (Fig. 4; SI Fig. S9). However, under our experimental conditions, the TLR2 deficiency appeared permissive for a more prominent modulation of IH-induced inflammatory stress response via neuroplastin in distinct hippocampal DG *stratum* *granulare* (*t* test for independent samples, *t* = − 2.72; df = 9; *P* = 0.023) and *moleculare* (*t* = − 3.09; df = 9; *P* = 0.013), and in CA2 region *stratum* *pyramidale* (*t* = − 2.71; df = 9; *P* = 0.024) and *stratum* *oriens* (*t* = − 2.61; df = 9; *P* = 0.028) (SI Fig. S9; also see SI Table S3).

### TLR2-induced neuroinflammatory response is localised to brain derived neurotrophic factor, neuroplastin and fibronectin-1-rich neurocircuitry

**Figure 5:**
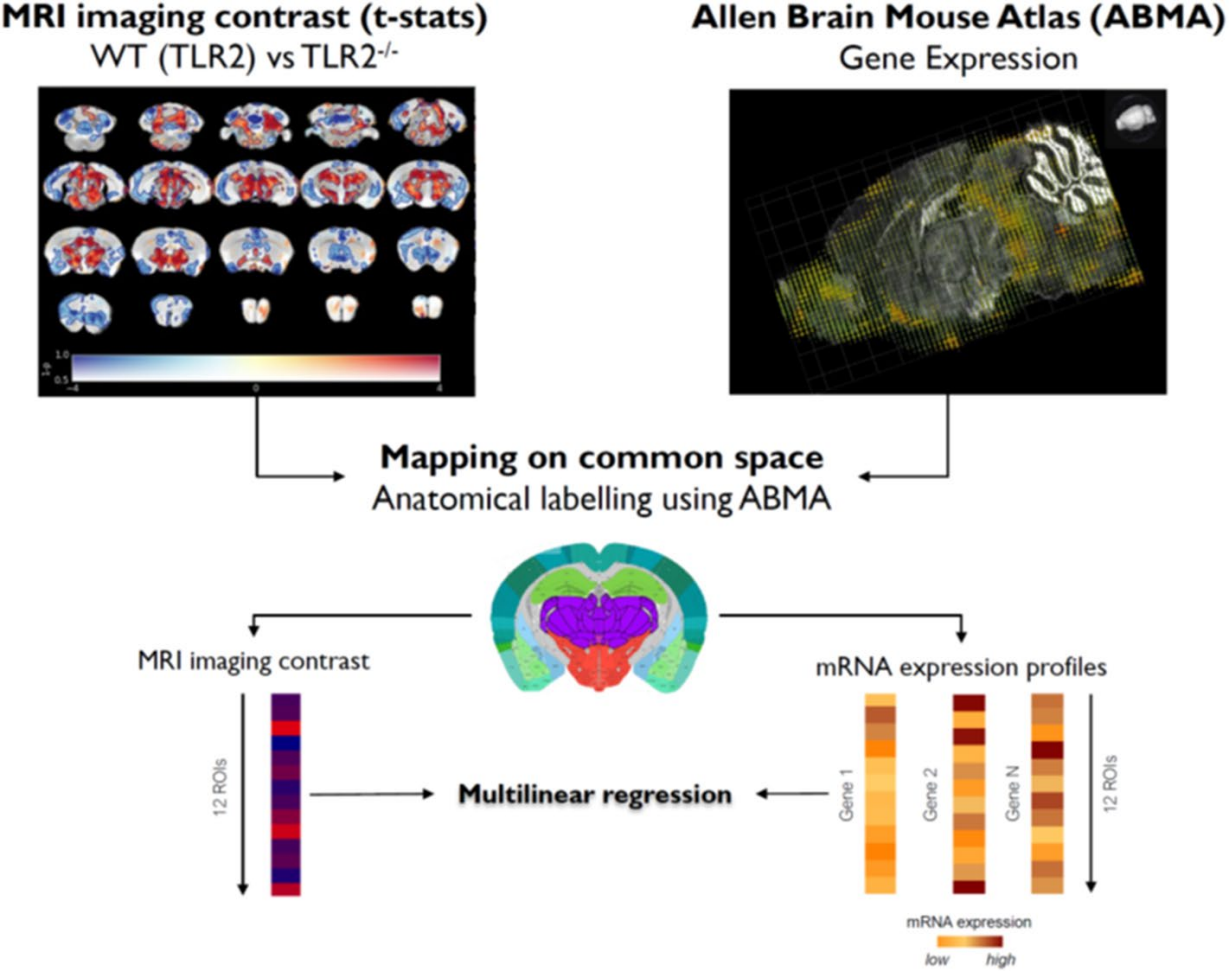
Schematic presentation of the MR-gene mapping research protocol: imaging *versus* mRNA expression analysis pipeline is shown. Effect of genotype (shown top left): voxel-wise differences in brain volume between TRL2^+/+^ and TLR2^−/−^ mice. Data are shown as dual-coded statistical maps in which volume change is coded by colour hue, and the family-wise error (FWE) corrected *P* values are coded by transparency. Warm (red) colours indicate larger volume in TLR2^*−/−*^ mice. Areas in which FWE-corrected *P* < 0.05 are contoured in black. Non-parametric statistics were performed using FSL randomize with 5,000 permutations and threshold-free cluster enhancement.

**Figure 6:**
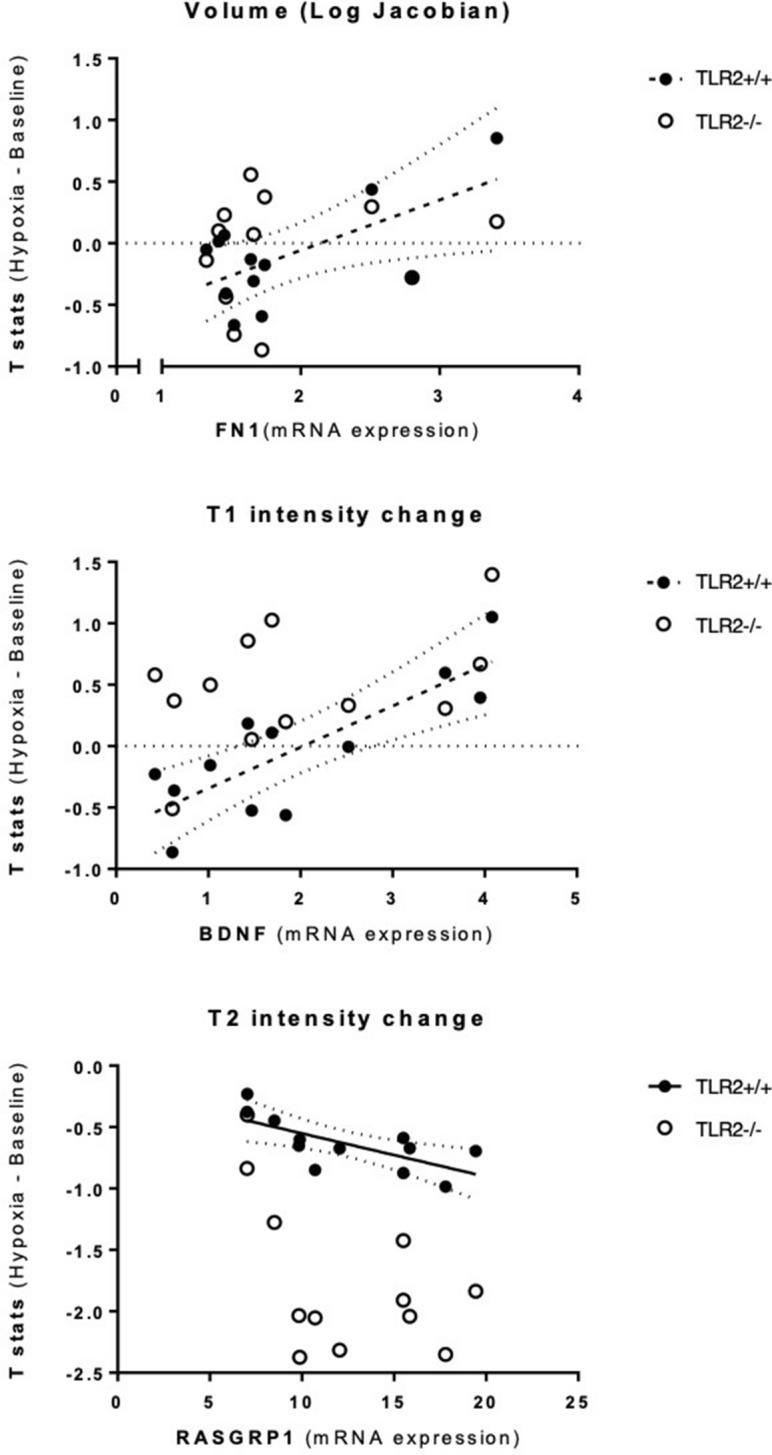
MRI imaging and gene expression correlation. All the correlations with TRL2^+/+^ were found to be statistically significant. All the correlation with TLR2^−/−^ were reported as not significant. *ABMA* Allen Brain Mice Atlas, *TLR2* Toll like receptor 2.

Taken together, we demonstrated the role for TLR2 in modulation of the neuroinflammatory response in a distinct brain circuitry. We then further considered the molecular origins that might underlie mechanisms behind observed changes. To this end, an investigation of the observed network’s modulation in mice with (TLR2^+/+^) and without functional TLR2 (TLR2^−/−^) was undertaken by linking it with the microregional brain plasticity gene expression profiles (Fig. 5; see SI Table S5). For this purpose the *Allen* *Brain* *Mouse* *Atlas*^23^ mRNA gene profile expression database and MR parametric *t*-statistic maps were utilised via a novel mapping method, recently described^24^. Several major neuroplasticity genes were initially found to show a strong association, but only in mice with a functional TLR2-system (SI Table S4). The genes that were linked with TLR2-enabled structural network reorganisations were the brain derived neurotrophic factor (BDNF), neuroplastin, Calcium/calmodulin dependent protein kinase II alpha (CAMK2A), Rho Guanine Nucleotide Exchange Factor 6 (ARHGEF6), Cholecystokinin (CCK), Fibronectin 1 (FN1) and RAS guanyl nucleotide-releasing protein 1 (RASGRP1) (SI Table S4). Further multiple regression analysis suggested the strongest, statistically significant permissive role for BDNF, FN1 and RASGRP1. A significant correlation of longitudinal relaxation time (T1) with BDNF expression was shown (r = 0.819; *P* = 0.002) (Fig. 6). FN1gene expression was predictive of brain volume increases (r = 0.627; *P* = 0.039). Conversely, RASGRP1 gene had an inverse relationship to transverse relaxation time (T2, r = − 0.646; *P* = 0.033) (Fig. 6), suggestive of its protective microregional role in topical inflammation. Strikingly, no links were demonstrated between any of the investigated neuroplasticity genes with any structural changes in TLR2 deficient mice (TLR2^−/−^).

### Neuroinflammatory response: from effects on mood, cognition to effects on weight gain

After describing distinct structural, cellular and molecular basis of the observed inflammatory response, we set out to explore its impact on behavioural changes. Specifically, we wanted to assess if mice in our OSA model shared similar behavioural and cognitive changes to those frequently reported in patients with OSA, including their well-documented struggles with obesity, somnolence, fragile mood and memory, and attention deficits^2,25^. We also wanted to see if those changes were linked to neuroanatomical ROIs that were initially highlighted by our BL and MR imaging: frontal cortex^14^, septal nuclei, ventral hippocampi^26^ and PAG^27,28^.

#### Weight 

**Figure 7:**
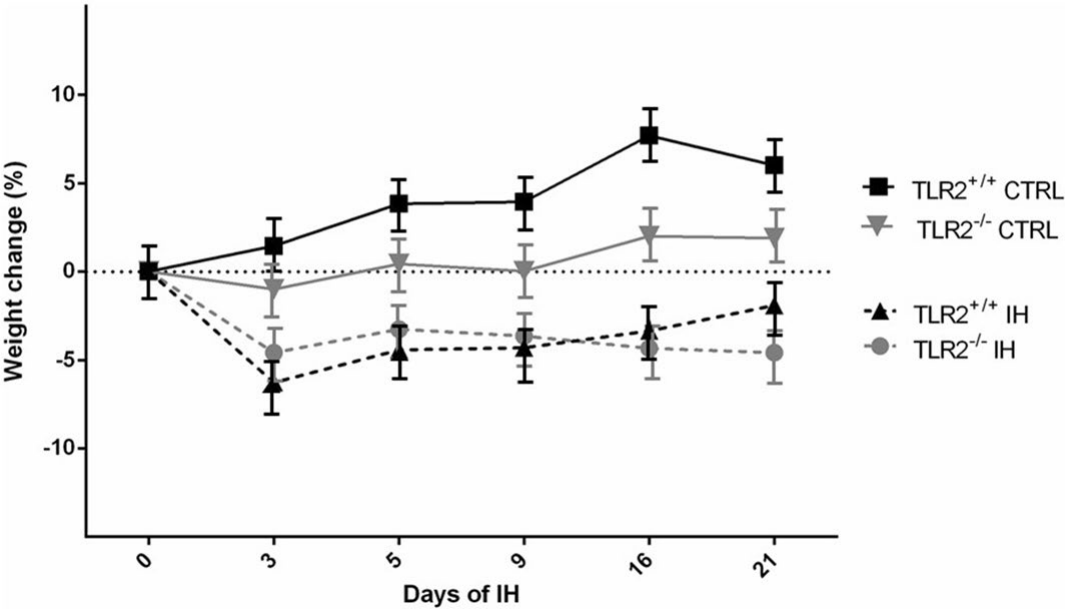
Significant body weight loss induced by intermittent hypoxia. Weight changes in four investigated groups are shown. IH protocol was associated with significant weight loss after 3 days in mice (e.g. TLR2^+/+^IH and TLR2^−/−^IH, F = 11.6, *P* < 0.001), irrespective of their TLR2 status. However, mice with functional TLR2 gene (TLR2^+/+^IH) were able to regain most of their lost weight by the end of the experimental period. Overall, having a functional TLR2 gene enabled weight gain, while deficient (TLR2^−/−^) mice struggled to regain weight. *Y* *axis* denotes time points (days) during the experimental protocol. Illustrative error bars shown (see SI Table S7 for nominal SD values). *CTRL* control, *IH* intermittent hypoxia protocol, *SD* standard deviation, *TLR2* Toll like receptor 2.

Firstly, the potential role for the TLR2 system in weight gain was investigated. In our study we demonstrated changes in two brain regions, which were recently proposed as anorexigenic in the neural circuitry of the rodents—the ventral hippocampus and lateral septal nucleus^26^. Specifically, structural comparisons demonstrated significant differences in the volumes of lateral septal nuclei [V(%): TLR2^+/+^IH 0.373 ± 0.007 vs TLR2^−/−^IH 0.335 ± 0.004; F = 24.97; *P* = 0.01] and in ventral hippocampi [V(%): TLR2^+/+^IH 0.0028 ± 0.0001 vs TLR2^−/−^H 0.00294 ± 0.00004; F = 8.80; *P* = 0.0002], suggested TLR2-driven changes under the inflammatory response. Similar structural differences were noted in control groups, although they were statistically remarkable only in the anorexigenic region of the right ventral hippocampi [V(%): TLR2^+/+^CTRL 0.00273 ± 0.00004 vs TLR2^−/−^CTRL 0.00308 ± 0.00007; F = 31.26; *P* = 0.0001]To further test if there is indeed a stronger baseline anorexigenic “structural” component in the neural circuit of TLR2-deficient knockout mice (TLR2^−/−^), we investigated the effect of TLR2 genotype on weight gain. In keeping with our structural data, a strong link between a functional TLR2 system and an ability to gain weight was demonstrated, both under control [TLR2^+/+^CTRL: third day (%): 1.17 ± 2.86; sixth day: 3.58 ± 2.39; ninth day: 6.25 ± 10.14;16th day: 7.64 ± 4.54; 21st day: 5.60 ± 4.34] and under IH experimental conditions [TLR2^+/+^IH: third day (%): − 6.28 ± 5.43; sixth day: − 4.43 ± 4.49; ninth day: − 4.31 ± 5.04; 16th day: − 3.35 ± 6.1; 21st day:  − 1.99 ± 6.05) (Fig. 7). Although exposure to IH resulted in significant weight loss in both TLR2^+/+^IH and TLR2^−/−^IH mice, compared to their respective controls (Fig. 7, Supplement), TLR2^−/−^ deficient mice (e.g. both TLR2 TLR2^−/−^IH and TLR2^−/−^CTRL) overall struggled to gain weight over the period of 3 weeks (Fig. 7). Thus, while mice with functional TLR2 (TLR2^+/+^) showed a steady state increase in weight, even following the initial loss under IH, TLR2^−/−^ mice were not able to regain initially lost weight (Fig. 7). This finding is highly suggestive of a link between TLR2-system, neuroinflammation and the weight gain in OSA.

#### Mood and cognition

Several behavioural tests (SI Table S6) known to assess and target psychomotor changes, affective and cognitive symptoms were used to investigate the role of TLR2 in neurocognitive changes. We observed two primary findings, which were consistently replicated across several behavioural tests and their parameters (SI Table S6). Firstly, as expected, we demonstrated increased psychomotor agitation and anxiety in all mice exposed to the IH protocol. This behaviour appeared independent of their TLR2-functionality. For example, during the tail suspension test (TST), the mice that were exposed to the experimental IH protocol spent significantly more time trying to escape the uncomfortable setting, and less time being immobile (immobility period:TLR2^+/+^IH: 103.72 ± 48.09 s; n = 15; TLR2^−/−^IH: 99.43 ± 33.17 s; n = 16) compared to their respective control groups (TLR2^+/+^CTRL: Z = − 1.95, *P* = 0.051; 152.68 ± 34.69 s, n = 12; TLR2^−/−^CTRL: Z = − 2.45, *P* = 0.014; 144.90 ± 56.08 s, n = 8).

The second finding was unexpected. It was noted that functional TLR2 genotype in mice exposed to IH protocol was linked to a specific “proactive” behavioural endophenotype. This was, for example, demonstrated by shorter latencies to trying to first escape during the TST (TST: TLR2^+/+^IH27.65 ± 7.11 s; TLR2^+/+^CTRL 27.37 ± 12.79 s vs TLR2^−/−^IH 45.04 ± 22.08 s; TLR2^−/−^CTRL 30.14 ± 12.38 s). Here functional TLR2 genotype appeared to rescue depression-like behaviour noted under experimental IH conditions of repeated stress (Z = − 2.12,*P*_(TLR2_^+/+^_IH vs TLR2_^−/−^_IH)_ = 0.027). We then investigated which ROIs might have functionally contributed to this behavioural endophenotype in TLR2^+/+^IH mice. Correlation analyses suggested significant divergent link to (left) frontal cortical (*P* = 0.02, r = 0.635) and periaqueductal grey region (*P* = 0.083, r = − 0.499; also see SI Table S1). A significant aberrant connectivity between the two regions was also noted (*P* = 0.011; r = − 0.66).

In regard to other behavioural findings, only a statistical trend for more effective spatial navigation and cognitive processing was seen in mice with functional TLR2 (TLR2^+/+^). For example, in the Y-maze test, the presence of the TLR2 system appeared to partially rescue deficits in spatial acquisition following exposure to IH, otherwise recorded in TLR2^−/−^ mice, as measured by the path efficiency (path efficiency%: TLR2^+/+^IH1.05 ± 0.16; TLR2^+/+^CTRL1.09 ± 0.20; TLR2^−/−^IH0.63 ± 0.14; TLR2^−/−^CTRL1.04 ± 0.20; Z = − 1.82, *P*_(TLR2_^+/+^_IH vs TLR2_^−/−^_IH)_ = 0.06) (see SI Table S6 for further details).

## Discussion

Whilst it has been accepted that OSA promotes a low-grade chronic systemic inflammation^29^, it has been a matter of some significant speculation whether it may also cause neuroinflammation^2^. We here advance that OSA may indeed promote a significant inflammatory response in the brain, that can result in specific structural and behavioural changes. To the best of our knowledge, this is the first direct demonstration of neuroinflammatory response under OSA conditions. We demonstrate that inflammatory spread occurs in the associated distinct neurocircuitry, and notably we report subsequent structural changes that closely correspond those previously recorded in clinical studies of patients with OSA^2,17,30^. Moreover, a clear link with structural changes and further functional and metabolic alterations is suggested by our findings, with possible significant translational clinical implications. More specifically, TLR2 system-driven changes in fronto-brainstem and hippocampal-septal circuitry are demonstrated, with links to agitated behaviour under episodes of stress, and an increased ability to gain weight.

Patients with OSA are known to be prone to obesity^31^, they have well documented cognitive and neuropsychiatric deficits^25^, excessive daytime somnolence, and are more likely to develop depression and anxiety^3,25,30^. They are also known to be particularly prone to traffic and general work accidents, in part through their well-documented deficits in attention^25^, but also presumably through the erroneous encoding of spatial information in the context of navigation^2,32^. Somewhat surprisingly, our data suggests an early antidepressogenic effect of the neuroinflammatory response in OSA that is TLR2-dependent, and which is functionally linked to a distinct fronto-brainstem subcircuitry. The activation of a similar network in mice has been reported to favour effortful behavioural responses to challenging situations^33^. More specifically, it was shown that a selective activation of a subclass of prefrontal cortex cells, which project to the brainstem, has been able to induce a profound, rapid and reversible effect on selection of the active behavioural states^33^. According to a description of traditional learned-helplessness response^34^, depressed patients do not favour effortful behavioural responses to challenging situations. To the contrary they are, through their own overvalued negative evaluations of challenges, significantly more likely to earlier retire from trying to find solutions out of their predicament. Conversely, in our study, mice demonstrate a clear, at least initially adaptive, ‘hyperactive’ behavioural response when placed in the challenging situation. They, unlike their TLR2-deficient counterparts, continued to try to find solutions out of their predicament. It would be of paramount interest to see if this initial adaptive behavioural response with time develops into a potentially more dangerous, futile, and energy wasting ‘agitated’ depressive profile^34,35^, with heightened anxiety component. A similar mixed anxiety and depression endophenotype has indeed been previously described in some patients with OSA^36^, and it has been traditionally linked with higher suicide risks in depressed patients^37^. Strikingly, only recently, in post-mortem brains of depressed patients who committed suicide, TLR2-protein and its mRNA gene expression have been shown significantly increased, especially in their frontal cortical regions^11^. Hence, an early induction of the neuroinflammatory process via TLR2-system in frontal regions and basal forebrain, such as was demonstrated in our mice, may be of particular importance in understanding the neural circuitry underlying pathological behavioural patterns of action selection and motivation in behaviour of patients with OSA^3^.

Our data suggests a potential modulatory role to the neuroinflammatory response in regards to feeding^35^ and or ability to gain weight. OSA has been associated with significant metabolic changes, diabetes and weight gain, thought to be at least partly modulated through its systemic inflammatory effects^4^. It has been previously proposed that a significant interplay between central nervous inflammation and systemic inflammation might occur in affected patients, which then further modulates bidirectional links between metabolism, weight gain and neurological disorders^12^. More specifically, our results could be taken to propose that activated TLR2-system enables continued feeding drive under conditions of repeated stress (Fig. 7). Presumably, similar drive could later cause maladaptive inability to control one’s food intake and to lead to an increased link between our emotional states and eating, causing an excessive weight gain in patients with OSA. The feeding circuitry that controls emotional or cognitive aspects of food intake is still largely unknown. However, recently, Sweeney and Yang^26^ demonstrated an anorexigenic neural circuit originating from ventral hippocampus to lateral septal nuclei in the brain, revealing a potential therapeutic target for the treatment of anorexia or other appetite disorders. Interestingly, we were able to differentiate this network solely based on the functionality of the TLR2-system in our mice, and severity of changes appeared further exacerbated by the IH stress (also see SI Table S1 for further details). Whilst preliminary, these findings might go some way to suggest an aberrant feeding circuit that controls emotional and cognitive aspects of food intake in patients with OSA.

In conclusion, utilising the rodent model of OSA that verifiably replicates arousals and hypoxaemia in patients with OSA^18^ and visualising in vivo TLR2-activation, we were able to demonstrate early activation of microglia in regions of basal forebrain, with later widespread frontal projections, suggesting a pivotal role for TLR2 in brain’s response to OSA injury. Our results provide the first in vivo evidence of an OSA-induced inflammatory response, with septal nuclei, the major source of cholinergic input to the hippocampus, being highlighted as a region of particular vulnerability early on in the neuroinflammatory process (Fig. 1C). Moreover, we were also able to link the initial neuroinflammatory response to later hypotrophic and hypertrophic neuroanatomical changes, with potential primary molecular drivers, such are neuroplastin and BDNF, also highlighted by our results. Through these findings, a fingerprint of a distinct OSA-effected neurocircuitry has emerged, with frontal regions and septal nuclei being suggested as an initial TLR2-dependant seed sites.

Our neuroimaging results are in broad agreement with previous findings in patients with OSA where compensatory mechanisms, activation of various homeostatic gene programmes and astroglial neurogenesis have all been proposed to underlie some of the initial functional and later maladaptive changes^17,38^. In further agreement, our data also suggests that differential plastic response to OSA in the brain may depend on regional genes expressional profiles^39^ For example our ‘MR-gene’ mapping findings support permissive and cohesive role for the TLR2-system in the interplay with BDNF, RSGRP1, fibronectin and neuroplastin-driven significant discrete and transformative neurophysiologic and behavioural changes.

Of the listed genes, the enhanced BDNF-levels have long been associated with increased plasticity and promotion of a growth-permissive environment, and its depletion with many neurological and psychiatric disorders^40^. The concept of the interplay between BDNF and TLR2-system in our study might also explain (preconditioning) protective effect on visuo-spatial memory in TLR2^+/+^IH mice, which, however, showed only significant trend. BDNF has been shown to enhance synaptic plasticity and neuron function in response to physical activity, learning and memory, and its baseline expression and activity-dependent upregulation in the hippocampus is believed to be under control of the medial septal nuclei, and important ROI in our study, with an important regulatory role in REM sleep (Fig. 1, SI Fig. S8). Patients with OSA show a rapid decrease in serum and plasma BDNF levels during initiation of the treatment [with positive airway pressure (PAP)-device], likely reflecting enhanced neuronal demand for BDNF in this condition^41^. Similarly, OSA patients had increased TLR2-expressions on blood immune cells, which could be reversed with PAP treatment^42^. TLR2-deficiency has been shown to impair neurogenesis previously^43^ and more recently, the TLR2-receptor has been shown to enhance adult neurogenesis in the hippocampal DG after cerebral ischaemia^44^. Of particular note to our findings, the loss of TLR2 has been recently shown to abolish repeated social defeat stress-induced social avoidance and anxiety in mice, and its deficiency mitigated stress-induced neuronal response attenuation, dendritic atrophy, and microglial activation in the medial prefrontal cortex^14^. TLR2 has also been shown to modulate inflammatory response caused by cerebral ischemia and reperfusion via linking to endogenous ligands, such as fibronectin^45^. Fibronectin, on the other hand, has been shown to act as a reparative molecule that promotes cellular growth and its levels are enhanced after brain injury in variety of disorders, including the AD^40,46^. Finally, we have been able to show significantly higher levels of the cell adhesion molecule neuroplastin in mice with functional TLR2-system. This is of note as neuroplastin is known to play a role in synaptic plasticity (e.g. long-term potentiation), formation and a balance of the excitatory/inhibitory synapses^47^, in shaping of brain’s cortical thickness^48^. Moreover, its involvement in early tissue response in hippocampi of AD patients has also been recently shown^49^. Interestingly, in TLR2-deficient mice, IH resulted in more prominent upregulation of this molecule (SI Fig. S9B), perhaps suggesting that in the absence or insufficient TLR2 response, neuroplastin might play a more prominent regulatory role during the inflammatory response. It is tempting to postulate that in patients with comorbid AD and OSA, and or in those with impaired TLR2 microglial response, any such compensatory effect could lead to imbalance in excitatory/inhibitory synapses at the hippocampal level, with serious consequences.

Taken together, the implicated gene programmes also indirectly suggest that modulations in neurites, cytoskeletal and receptor signalling, cell adhesion, axonal sprouting and other extracellular and perineuronal nets likely underlie the observed structural and functional changes. Whilst our findings are striking and theory-forming, our study leaves many questions still unanswered. There are several limitations to our findings behind OSA-injury, notwithstanding that the majority of experiments were done in cross-sectional manner, preventing us from deduction of causality and or direction of noted changes. Ideally, multimodal longitudinal in vivo manipulation of the microglia TLR2 in the highlighted ROIs, along with in vivo functional MR and further behavioural, genetic and electroencephalographic studies, should help shed much needed insight into here proposed novel neural mechanisms. Nonetheless, we believe that the distinct regional association between highlighted gene profiles and structural and functional changes, as demonstrated by our data, arguably further indicates a true circuitry-specific, rather than wider systemic, nature of TLR2-modulated neuroinflammatory injury and as such provide a plethora of potential investigational targets for future studies.

## Methods

Two mouse lines were used, C57BL/6-Tyr^c-Brd^-Tg(Tlr2-luc/gfp)^Kri^/Gaj and C57BL/6-*Tlr2*^*tm1Kir*^, (further in the text TLR2^+/+^ and TLR2^−/−^ respectively), both previously described by our collaborators^20^ (see Supplemental Material and Methods). Four experimental groups were compared: group 1 (TLR2^+/+^IH), mice with functional TLR2 system that were exposed to 3 weeks of chronic IH protocol^19,22^; group 2 (TLR2^+/+^CTRL), mice with functional TLR2-system that were handled under control (CTRL) conditions; group 3 (TLR2^−/−^IH), TLR2 knock out mice exposed to 3 weeks of chronic IH; and group 4 (TLR2^−/−^ CTRL) control TLR2 knock out mice. In BLI experiments we compared 2 groups (TLR2^+/+^IH and TLR2^+/+^CTRL) of mice, and all other investigations were done between 4 groups (TLR2^+/+^IH, TLR2^+/+^CTRL, TLR2^−/−^IH and TLR2^−/−^CTRL) (SI Figure S1 depicts research protocol). All investigations and animal interventions were approved by the Ethics Committee of the University of Zagreb, School of Medicine and experiments were performed in accordance with relevant guidelines and regulations.

### Behavioural tests

Open field test (OF): to determine the spontaneous horizontal locomotor activity, an open-field test was performed, and all the test parameters calculated, as previously described^50^. *Y-*maze test (YM): To assess the working memory, a Y-maze test was conducted and all the test parameters calculated, as previously described^50^. Tail suspension test (TST): the TST was performed to evaluate depression-like behaviour. Scoring of immobility time was performed by means of automated video tracking software, as described in detail previously^51^.

### MRI acquisition

MR images were acquired on a 7 T scanner (Agilent). High resolution quantitative T1 and T2 maps were acquired using a modified DESPOT1 and DESPOT2-FM protocol^52^. This consisted of Spoiled-Gradient-Recalled (SPGR) images with TE/TR = 14.6/32 ms, readout bandwidth 10 kHz and seven flip-angles (5°–35° in 5° steps) and balanced Steady-State-Free-Precession (bSSFP) images with TE/TR = 4/8 ms, readout bandwidth 62.5 kHz, seven flip angles (8°, 12°, 16°, 24°, 32°, 40° and 48°) and four phase increments (45°, 135°, 225° and 315°). The flip-angles were chosen to lie between the optimum values for the expected values of T1 and T2^53^ , which were obtained from a similar preparatory scan. An actual flip-angle-imaging (AFI) scan was acquired for B1 inhomogeneity correction at matrix size 96 × 96 × 96, 333 μM isotropic voxel sizes, TE/TR1/TR2 = 6.52/20/100 ms, readout bandwidth 10 kHz and flip-angle 55°^54,55^**.**

### MRI and statistical analyses

The MR images were processed using a combination of FSL^56^, ANTs^57^ and the QUIT toolbox^58^, as previously described by our group^59^. A group analysis was carried out on Jacobian determinant images with permutation tests and threshold-free cluster enhancement (TFCE) using FSL randomize^60,61^. Data were displayed on the mouse template image, using the dual coding approach^62^: differences were mapped to color hue, and associated t-statistics were mapped to color transparency. Contours were family wise error (FWE) corrected statistically (P < 0.05) significant differences.

The Kolmogorov–Smirnov test was used to test the normality of distributions. In addition, for all variables that were normally distributed one-way analysis of variance and Bonferroni corrections were used, as previously described^38^. Pearson correlation coefficients were calculated between all investigated variables and used to determine heatmaps. Wilcoxon test was used for comparison BLI (photon emissions) between each two measures, and for the analysis of the behavioral tests. *T* test for independent samples was used to analyze differences in neuroplastin immunoreactivity. All statistical analyses had a two-tailed α level of < 0.05 for defining significance and were performed by an experienced biostatistician (M.M.) on the statistical software IBM SPSS Statistics version 23 (www.spss.com).

Neuroplastin immunoreactivity^47^, perfusion and histology for MRI^21^ were all performed as previously described in detail by our group. Further methodological description of mice lines, experimental and study procedures, including the undertaken statistical and MR analyses, is additionally available in the Supplement.

## Supplementary information

Supplementary information

## Data Availability

The data that support the findings of this study are available from the corresponding author upon reasonable request.
