# Supplementary material for: The innate immune toll-like-receptor-2 modulates the depressogenic and anorexiolytic neuroinflammatory response in obstructive sleep apnoea
Source: Sci Rep. 2020 Jul 10;10:11475. doi: 10.1038/s41598-020-68299-2 (PMC7351955; doi:10.1038/s41598-020-68299-2)
Supplement: Supplementary file 1 — Supplementary information [file 41598_2020_68299_MOESM1_ESM.docx]

# Supplement to: The Innate Immune Toll-Like Receptor-2 modulates the Depressogenic and Anorexiolytic Neuroinflammatory Response in Obstructive Sleep Apnoea

Dora Polsek, Diana Cash, Mattia Veronese, Katarina Ilic, Tobias Wood, Milan Milosevic, Svjetlana Kalanj-Bognar, Mary J. Morrell, Steve C.R. Williams, Srecko Gajovic, Guy D. Leschziner, Dinko Mitrecic, Ivana Rosenzweig.

**Running Title**: Sleep Apnoea and Neuroinflammation

## Supplement Outline

1. Materials and Methods

- Figure S1 – Research Protocol

2. Structural Neuroimaging Changes

- Figures S2-4

- Table S1

3. Morphologic and Cellular Changes

- Figure S5-8

- Tables S2, 3

4. Integration of Structural Neuroimaging and mRNA Brain Expression Maps

- Tables S4,5

5. Behavioural Changes

-Table S6

6. Weight Changes

- Tables S7,8

*References*

## Materials and Methods

### Animals: For this study two mouse lines were used, C57BL/6-Tyr^c-Brd^-Tg(Tlr2-luc/gfp)^Kri^/Gaj and C57BL/6-Tlr2^tm1Kir^, named in the text as TLR2^+/+^ and TLR2^-/-^ respectively. C57BL/6-Tyr^c-Brd^-Tg(Tlr2-luc/gfp)^Kri^/Gaj mouse line was generated in two steps. Firstly, the transgenic mouse model was generated using a Tlr2 promotor and bicistronic luc/gfp transporter on a C57BL/6 genetic background, as previously described.^1^ The line was backcrossed to a C57BL/6-Tyr^c-Brd^ mouse background with the goal of enhancing the capture and precision of analysis of the bioluminescent signal in the albino C57BL/6 mice which have a mutation in the tyrosinase gene that enables the photons to penetrate better through the fur of the animal. The second mouse line used was C57BL/6-Tlr2^tm1Kir^, a C57BL/6 mouse line with the Tlr2 gene knocked out on both alleles (Jackson Laboratory, Maine, USA). All the mice used were male, 2-4 months old, bred at the animal facility at the Croatian Institute for Brain Research. The day/ night cycle was defined as 12h -12h, from 7 a.m. to 7 p.m. Food and water were available ad libitum. The animals were housed in transparent polysulphane cages of European standard type 3 (1290D, Tecniplast, Italy). Ten mice were housed in each cage, with the bedding changed every two days.

### Experimental Design: All animal experiments were done with the permission of the Ethics committee of the University of Zagreb, School of Medicine, ethical permit no. 380-59-10106-14-55/230.

####
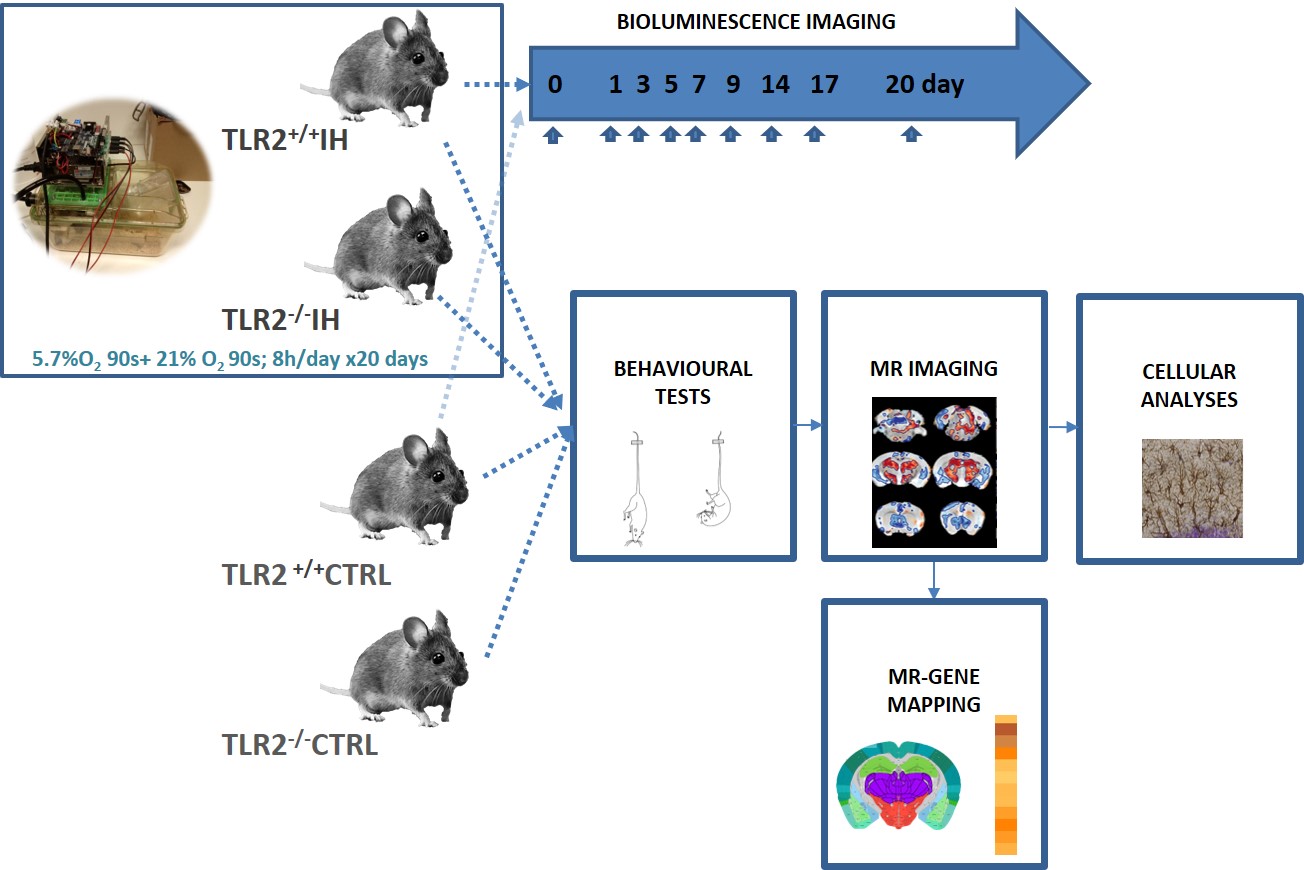


#### Figure S1 Schematic presentation of the research protocol. Four experimental groups were compared in this study: TLR2^+/+^IH (C57BL/6-Tyr^c-2J^(Cg)-Tg(Tlr2-luc/gfp) 275S Kri), mice with functional TLR2 system that were exposed to three weeks of chronic IH protocol; TLR2^+/+^CTRL (also C57BL/6-Tyr^c-2J^(Cg)-Tg(Tlr2-luc/gfp) 275S Kri), mice with functional TLR2 system that were handled under control conditions; TLR2^-/-^ IH (C57BL/6-Tlr2^tm1Kir^), TLR2 knock out mice exposed to three weeks of chronic IH; and TLR2^-/-^ CTRL (also C57BL/6-Tlr2^tm1Kir^) control TLR2 knock out mice. Adapted from^2, 3^.

### Perfusion and Histology For MRI: The mice were killed by transcardiac perfusion with ice-cold heparinized saline followed by 4% buffered paraformaldehyde (PFA). The heads were removed, stored in paraformaldehyde (PFA) for 24 hours and then rehydrated in Phosphate Buffered Saline (PBS) with 0.05% sodium azide at -4ºC for a minimum of 30 days^4^ .

The brains were sectioned at 20µm in a series of twelve, of which one was stained free floating with respective (e.g. iba1; cFos; GFAP; MBP; also please see Table S2) antibody to mark microglial, neuronal, astrocytic cells and myelin sheaths respectively. Once stained and mounted onto slides, the sections were scanned at x40 magnification on a *Leica SCN400F* slide scanner. Analysis of the respective positive cells was conducted using the optical fractionator method, as previously described^5^. To this end, combination of Matlab (mathworks.com) script for systematic random sampling of jpeg images from manually drawn ROIs (300µm x 300µm grid), and a stereological tool STEPanizer (Stepanizer.com)^6^ was used as previously published. A counting frame of 50µm x 50µm, to count iba1^+^ cells in jpegs according to the principles of unbiased stereological estimation^5, 7^, with Gundersen’s coefficient of error (CE) of 0.1 or above as an exclusion criterion.

cFos, GFAP and MBP staining (see Table S2) was analyzed by the thresholding method, as previously described by our group and others^8, 9, 10^. Briefly, using an open source *Aperio Image Scope* image viewer, the ROI were set at 4x magnification and a trichromatic image was generated with predefined thresholds for color saturation. The images were subsequently converted to eight-bit *BW* images and analyzed using *ImageJ* (1.51m9; https://imagej.net/ImageJ). The surface area fraction was generated, as percentage of pixels in the image that remained after aplying *Image J's* Huang threshold and background substraction, as previously shown^8, 9^.

***Behavioural tests:*** *Open Field test* (OF): To determine the spontaneous horizontal locomotor activity, an open-field test was performed, and all the test parameters calculated, as previously described^11^. The test was carried out in clear black Plexiglas boxes (40 × 40 × 40 cm) equipped with the video-based system. The test box was cleaned with 70% ethanol between each test. *Y-maze test* (YM): To assess the working memory, a Y-maze test was conducted and all the test parameters calculated, as previously described^11^. Arms were cleaned with 70% ethanol between each test to remove odours and residues. The alternation score (%) for each mouse was defined as the ratio of the actual number of alternations to the possible number (defined as the total number of arm entries minus two) multiplied by 100 as shown by the following equation: % Alternation = (Number of alternations)/(Total arm entries - 2) × 100. The number of arm entries was used as an indicator of locomotor activity.^11^ *Tail Suspension test* (TST): The TST was performed to evaluate depression-like behaviour. We recorded the overall time that animals were immobile while suspended by the tail over a 6 min period. Scoring of immobility time was performed by means of automated video tracking software as described previously.^12^

### MRI acquistion: MR images were acquired on a 7 Tesla scanner (Agilent). Samples were immersed in fluorinated liquid to reduce susceptibility artefacts (Galden; Solvay) and loaded four at a time into a 39 mm diameter transmit-receive birdcage coil (Rapid GmbH). High resolution quantitative T1 and T2 maps were acquired using a modified DESPOT1 and DESPOT2-FM protocol^13^. This consisted of Spoiled Gradient Recalled (SPGR) images with TE/TR=14.6/32 ms, readout bandwidth 10kHz and seven flip-angles (5-35 degrees in 5 degree steps) and balanced Steady-State Free Precession (bSSFP) images with TE/TR = 4/8 ms, readout bandwidth 62.5 kHz, seven flip angles (8,12,16,24,32,40 & 48 degrees) and four phase increments (45,135,225 and 315 degrees). The flip-angles were chosen to lie between the optimum values for the expected values of T1&T2^14^ , which were obtained from a similar preparatory scan. Both SPGR and SSFP had 256x256x256 matrix size with 125 micron isotropic voxels. An Actual Flip-angle Imaging (AFI) scan was acquired for B1 inhomogeneity correction at matrix size 96x96x96, 333 micron isotropic voxel sizes, TE/TR1/TR2 = 6.52/20/100 ms, readout bandwidth 10 kHz and flip-angle 55 degrees^15, 16^

***MRI analysis:*** The MR images were first converted to NIFTI format and then processed using a combination of FSL^17^, ANTs^18^ and the QUIT toolbox^19^. The processing pipeline consisted of several steps, described in detail previously by our group^20^. Briefly, images were Tukey filtered in k-space, and then B1, T1 & T2 maps calculated from the AFI, SPGR and SSFP scans respectively. Finally, a synthetic Spin Echo image was calculated from the T1 & T2 maps with TE/TR = 40/10000 ms for registration purposes. This created an almost purely T2-weighted image that matched the contrast of a widely available atlas image^21^.The combined 4 images were split into individual subjects and rigidly registered to the atlas to ensure approximate alignment. A template image was then constructed from all subjects in the study^18^, which was non-linearly registered to the atlas image. Logarithmic Jacobian determinants were calculated from the inverse warp fields in standard space to estimate apparent volume change. The combined transforms from native to atlas space were applied to all T1 and T2 maps, which were then smoothed with a Gaussian (150 micron FWHM). A brain parenchyma mask was created from the atlas labels by excluding cerebrospinal fluid (CSF) regions. The inverse of the combined transforms for each subject was applied to the atlas mask, and the resulting subject-specific masks were used to calculate the brain and ROIs volume for each subject.

### A group analysis was then carried out on Jacobian determinant images with permutation tests and Threshold-Free Cluster Enhancement (TFCE) using FSL randomize^22, 23^ . The brain volume estimates were included as a regressor of no interest in the design matrix when analysing the Jacobian determinants.

Data are displayed on the mouse template image, using the dual coding approach^24^: differences are mapped to color hue, and associated t statistics are mapped to color transparency. Contours are family wise error (FWE) corrected statistically (*P*<.05) significant differences.

### Neuroplastin immunoreactivity: Coronal sections of 4 animals per each group (four groups were: 1) TLR2^+/+^CTRL, 2) TLR2^+/+^IH, 3) TLR^-/-^CTRL and 4) TLR^-/-^IH) were incubated with 1% hydrogen peroxide in PBS for 30 minutes. After washing, section were incubated in blocking solution (5% horse serum in PBS) for 2 hours at +4°C. Incubation with primary anti‐neuroplastin 65 antibody raised in goat (1:500, R&D Systems, AF5360, Minneapolis, Minnesota, USA) in blocking solution was performed at +4°C overnight, as previously described by our group^25^. Parallel sections incubated in blocking solution without primary antibody were used as negative controls^25^. Incubation in secondary anti‐goat antibody conjugated with horse‐radish peroxidase (1:10 000, Jackson ImmunoResearch Laboratories, West Grove, Pennsylvania, USA) in blocking solution was performed at RT for 2 hours. Diaminobenzidine (DAB) was used as an enhancement agent for immunoreactivity visualization. Signal was intensified with additional incubation with 0.4% CuSO4^25^. Sections were scanned using a high resolution scanner (Hamamatsu NanoZoomer C10730‐12). Signal intensities of Np immunoreactivity were quantified using ImageJ densitometric analysis (ImageJ, NIH public domain, ttps://imagej.nih.gov/ij/)^25^. Statistical analysis of total Np immunoreactivity was done by using the Student's t test. All statistics were performed by using IBM SPSS ver. 25 (IBM Analytics, New York, NY, USA) software^26^.

### All other statistical analyses had a 2-tailed α level of < .05 for defining significance and were performed by an experienced biostatistician(M.M.) on the statistical software IBM SPSS Statistics version 23 ([www.spss.com](http://www.spss.com)).

### Structural Neuroimaging Changes


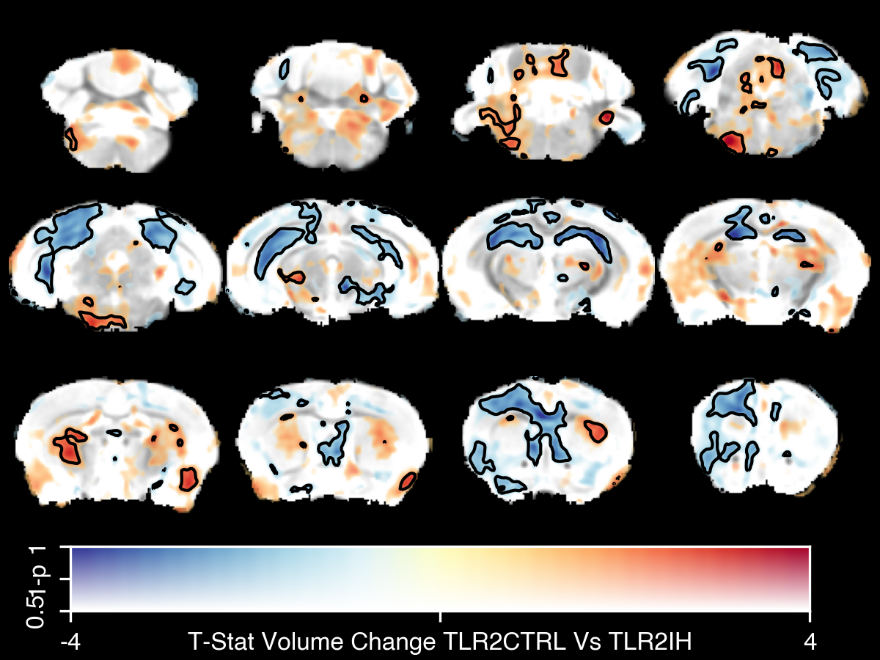


**A**


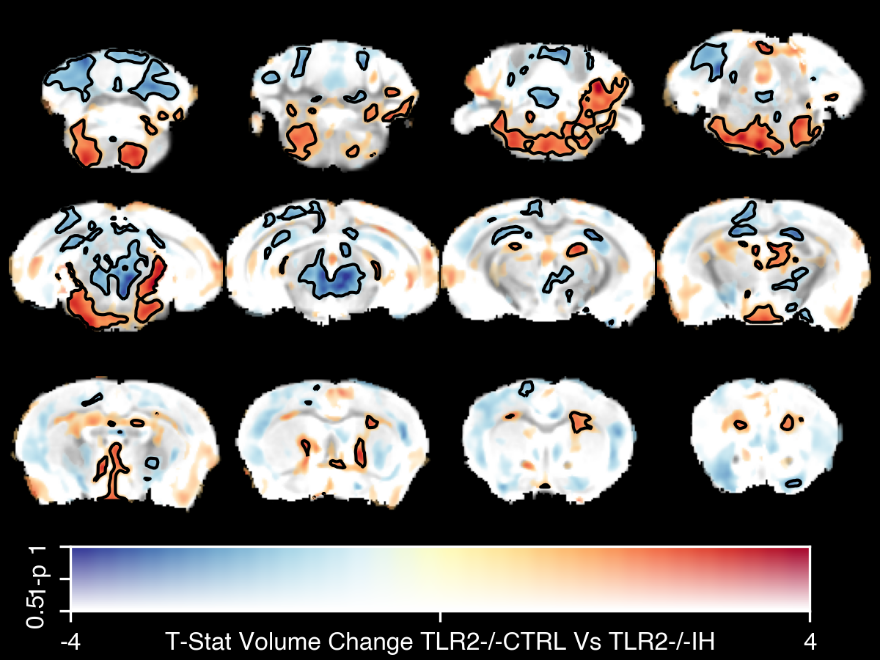


**B**

#### Figure S2 Differences in local brain volume between IH and CTRL conditions in TRL2^+/+^ (A) and TLR2^-/-^ (B) mice.

#### Data are shown as dual-coded statistical maps in which effect size is coded by color hue, and the family-wise error (FWE) corrected p-values are coded by transparency. Cold (blue) colors indicate larger volume in IH mice. Areas in which FWE-corrected P < .05 are contoured in black. Non-parametric statistics were performed using FSL randomize with 5000 permutations and threshold-free cluster enhancement. Hippocampus, left side of the motor and cingulate cortex and septum all appeared enlarged following IH, while these changes are not visible in TLR2 ^-/-^ mice. Reduction of volume after IH is visible in the reticular nuclei of the thalamus and dorsal striatum of TRL2^+/+^, and pronounced in pons, medulla and mesencephalon of TLR2 ^-/-^.

#### Table S1 Volumetric values of ROIs in grey and white matter in TLR2^+/+^IH (1; n=14), TLR^+/+^CTRL (2; n=11), TLR2^-/-^ IH (3; n=15), TLR2^-/-^CTRL (4; n=8). Values are given as percentage of total brain volume.

*Abbreviations***: Amy-** amygdala; **AC ant-** anterior commissure: pars anterior; **AC post-** anterior commissure: pars posterior; **AV-** arbor vita of cerebellum; **Bsl FB-** basal forebrain; **BNST-** bed nucleus of stria terminalis; **Cbl Ctx-** cerebellar cortex; **Inf Cb Ped-** cerebellar peduncle: inferior; **M Cb Ped-** cerebellar peduncle: middle; **Sup Cb Ped-** cerebellar peduncle: superior; **Cbr Aq-** cerebral aqueduct; **Enr Cx-** cerebral cortex: entorhinal cortex; **F Cx-** cerebral cortex: frontal lobe; **Occ Cx-** cerebral cortex: occipital lobe; **Ptemp Cx-** cerebral cortex: parieto-temporal lobe; **Cbr Ped-** cerebral peduncle; **Inf coll-** colliculus: inferior; **Sup Coll-** colliculus: superior; **CC-** corpus callosum; **CST-** corticospinal tract/pyramids; **Cuneate N-** cuneate nucleus; **DG-** dentate gyrus of hippocampus; **Fac N-** facial nerve (cranial nerve 7); **Fsc Rflx-** fasciculus retroflexus; **Fmb-** fimbria; **Fx-** fornix; **4^th^ Vtr-** fourth ventricle; **F Str-** fundus of striatum; **GP-** globus pallidus; **Hab-** habenular commissure; **Hippo-** hippocampus; **Hypo-** hypothalamus; **Inf Oli-** inferior olivary complex; **Int Cap-** internal capsule; **IntPed Nuc-** interpedunclar nucleus; **Lat Olf T-** lateral olfactory tract; **Lat Spt-** lateral septum; **Lat Vtr-** lateral ventricle; **Mamm-** mammillary bodies; **Mamm Tr-** mammilothalamic tract; **ML-** medial lemniscus/medial longitudinal fasciculus; **Med Spt-** medial septum; **Medulla-** medulla; **Midbrain-** midbrain; **Nac-** nucleus accumbens; **Olf Blb-** olfactory bulbs; **Olf Tub-** olfactory tubercle; **Opt Tr-** optic tract; **Pag-** periaqueductal grey; **Pons-** pons; **Pon N-** pontine nucleus; **Post Com-** posterior commissure; **Pre ParaSub-** pre-para subiculum; **SG Hippo-** stratum granulosum of hippocampus; **Stria M-** stria medullaris; **Stria T-** stria terminalis; **Str-** striatum; **Sub Ependy-** subependymale zone / rhinocele; **Sup Oli-** superior olivary complex; **Thal-** thalamus; **3^rd^ Vtr-** third ventricle; **vHippo** -ventral hippocampus (head); **VT dec-** ventral tegmental decussation.

|  | | **Mean** | **SD** |
| --- | --- | --- | --- |
| LACant | 1 | 0.159 | 0.008 |
|  | 2 | 0.154 | 0.008 |
|  | 3 | 0.147 | 0.010 |
|  | 4 | 0.152 | 0.010 |
| LACpost | 1 | 0.054 | 0.005 |
|  | 2 | 0.052 | 0.005 |
|  | 3 | 0.049 | 0.004 |
|  | 4 | 0.050 | 0.004 |
| LAmy | 1 | 1.740 | 0.060 |
|  | 2 | 1.760 | 0.048 |
|  | 3 | 1.655 | 0.071 |
|  | 4 | 1.664 | 0.038 |
| LAV | 1 | 0.991 | 0.036 |
|  | 2 | 0.983 | 0.075 |
|  | 3 | 1.030 | 0.046 |
|  | 4 | 1.034 | 0.034 |
| LBNST | 1 | 0.156 | 0.010 |
|  | 2 | 0.149 | 0.008 |
|  | 3 | 0.152 | 0.008 |
|  | 4 | 0.154 | 0.006 |
| LBsFB | 1 | 0.526 | 0.020 |
|  | 2 | 0.525 | 0.032 |
|  | 3 | 0.532 | 0.025 |
|  | 4 | 0.522 | 0.041 |
| LCbCtx | 1 | 5.198 | 0.111 |
|  | 2 | 5.184 | 0.180 |
|  | 3 | 5.227 | 0.138 |
|  | 4 | 5.224 | 0.240 |
| LCbrPed | 1 | 0.258 | 0.013 |
|  | 2 | 0.254 | 0.020 |
|  | 3 | 0.267 | 0.010 |
|  | 4 | 0.269 | 0.017 |
| LCC | 1 | 1.783 | 0.046 |
|  | 2 | 1.776 | 0.098 |
|  | 3 | 1.787 | 0.056 |
|  | 4 | 1.798 | 0.041 |
| LCST | 1 | 0.175 | 0.009 |
|  | 2 | 0.179 | 0.010 |
|  | 3 | 0.171 | 0.011 |
|  | 4 | 0.181 | 0.013 |
| LCuneateN | 1 | 0.029 | 0.002 |
|  | 2 | 0.032 | 0.003 |
|  | 3 | 0.030 | 0.004 |
|  | 4 | 0.029 | 0.002 |
| LDG | 1 | 0.419 | 0.021 |
|  | 2 | 0.399 | 0.021 |
|  | 3 | 0.437 | 0.028 |
|  | 4 | 0.427 | 0.021 |
| LEnRCx | 1 | 1.151 | 0.056 |
|  | 2 | 1.160 | 0.058 |
|  | 3 | 1.123 | 0.049 |
|  | 4 | 1.124 | 0.040 |
| LFCx | 1 | 4.698 | 0.187 |
|  | 2 | 4.657 | 0.123 |
|  | 3 | 4.574 | 0.175 |
|  | 4 | 4.481 | 0.168 |
| LFacN | 1 | 0.026 | 0.002 |
|  | 2 | 0.026 | 0.001 |
|  | 3 | 0.026 | 0.001 |
|  | 4 | 0.027 | 0.001 |
| LFscRFlx | 1 | 0.028 | 0.002 |
|  | 2 | 0.028 | 0.002 |
|  | 3 | 0.029 | 0.002 |
|  | 4 | 0.029 | 0.001 |
| LFmb | 1 | 0.303 | 0.015 |
|  | 2 | 0.311 | 0.016 |
|  | 3 | 0.320 | 0.018 |
|  | 4 | 0.322 | 0.016 |
| LFStr | 1 | 0.025 | 0.002 |
|  | 2 | 0.023 | 0.003 |
|  | 3 | 0.024 | 0.003 |
|  | 4 | 0.022 | 0.002 |
| LFx | 1 | 0.066 | 0.004 |
|  | 2 | 0.066 | 0.005 |
|  | 3 | 0.069 | 0.004 |
|  | 4 | 0.069 | 0.003 |
| LGP | 1 | 0.324 | 0.019 |
|  | 2 | 0.324 | 0.012 |
|  | 3 | 0.338 | 0.015 |
|  | 4 | 0.339 | 0.016 |
| LHab | 1 | 0.003 | 0.001 |
|  | 2 | 0.003 | 0.001 |
|  | 3 | 0.003 | 0.001 |
|  | 4 | 0.003 | 0.000 |
| LHippo | 1 | 2.251 | 0.085 |
|  | 2 | 2.154 | 0.125 |
|  | 3 | 2.242 | 0.077 |
|  | 4 | 2.219 | 0.083 |
| LvHippo | 1 | 0.2761 | 0.0186 |
|  | 2 | 0.2639 | 0.0140 |
|  | 3 | 0.2939 | 0.0134 |
|  | 4 | 0.2789 | 0.0191 |
| LHypo | 1 | 1.183 | 0.049 |
|  | 2 | 1.175 | 0.042 |
|  | 3 | 1.216 | 0.034 |
|  | 4 | 1.229 | 0.041 |
| LInfCbPed | 1 | 0.083 | 0.007 |
|  | 2 | 0.084 | 0.008 |
|  | 3 | 0.084 | 0.006 |
|  | 4 | 0.088 | 0.009 |
| LInfcoll | 1 | 0.614 | 0.021 |
|  | 2 | 0.613 | 0.026 |
|  | 3 | 0.634 | 0.029 |
|  | 4 | 0.622 | 0.017 |
| LInfOli | 1 | 0.048 | 0.003 |
|  | 2 | 0.046 | 0.004 |
|  | 3 | 0.045 | 0.005 |
|  | 4 | 0.051 | 0.008 |
| LIntC | 1 | 0.294 | 0.018 |
|  | 2 | 0.300 | 0.011 |
|  | 3 | 0.305 | 0.013 |
|  | 4 | 0.305 | 0.016 |
| LLatSpt | 1 | 0.373 | 0.025 |
|  | 2 | 0.345 | 0.027 |
|  | 3 | 0.335 | 0.016 |
|  | 4 | 0.337 | 0.018 |
| LLatVtr | 1 | 0.420 | 0.029 |
|  | 2 | 0.414 | 0.025 |
|  | 3 | 0.405 | 0.021 |
|  | 4 | 0.404 | 0.022 |
| LLatOlfT | 1 | 0.141 | 0.012 |
|  | 2 | 0.147 | 0.010 |
|  | 3 | 0.144 | 0.012 |
|  | 4 | 0.147 | 0.022 |
| LMCbPed | 1 | 0.139 | 0.007 |
|  | 2 | 0.137 | 0.010 |
|  | 3 | 0.141 | 0.007 |
|  | 4 | 0.146 | 0.006 |
| LMamm | 1 | 0.056 | 0.006 |
|  | 2 | 0.057 | 0.005 |
|  | 3 | 0.058 | 0.005 |
|  | 4 | 0.058 | 0.002 |
| LMammTr | 1 | 0.028 | 0.002 |
|  | 2 | 0.027 | 0.002 |
|  | 3 | 0.030 | 0.003 |
|  | 4 | 0.030 | 0.002 |
| LMedSpt | 1 | 0.122 | 0.010 |
|  | 2 | 0.123 | 0.010 |
|  | 3 | 0.119 | 0.007 |
|  | 4 | 0.117 | 0.010 |
| LML | 1 | 0.256 | 0.008 |
|  | 2 | 0.253 | 0.013 |
|  | 3 | 0.261 | 0.011 |
|  | 4 | 0.263 | 0.016 |
| LNac | 1 | 0.422 | 0.015 |
|  | 2 | 0.412 | 0.027 |
|  | 3 | 0.406 | 0.033 |
|  | 4 | 0.396 | 0.012 |
| LOccCx | 1 | 0.622 | 0.029 |
|  | 2 | 0.607 | 0.036 |
|  | 3 | 0.605 | 0.021 |
|  | 4 | 0.598 | 0.013 |
| LOlfBlb | 1 | 2.783 | 0.103 |
|  | 2 | 2.822 | 0.119 |
|  | 3 | 2.789 | 0.087 |
|  | 4 | 2.815 | 0.097 |
| LOptTr | 1 | 0.187 | 0.007 |
|  | 2 | 0.183 | 0.008 |
|  | 3 | 0.186 | 0.008 |
|  | 4 | 0.188 | 0.008 |
| LOlfTub | 1 | 0.388 | 0.025 |
|  | 2 | 0.386 | 0.029 |
|  | 3 | 0.387 | 0.024 |
|  | 4 | 0.384 | 0.032 |
| LPonN | 1 | 0.084 | 0.006 |
|  | 2 | 0.086 | 0.009 |
|  | 3 | 0.089 | 0.009 |
|  | 4 | 0.095 | 0.007 |
| LPreParaSub | 1 | 0.281 | 0.019 |
|  | 2 | 0.259 | 0.022 |
|  | 3 | 0.282 | 0.016 |
|  | 4 | 0.279 | 0.013 |
| LPtempCx | 1 | 8.023 | 0.184 |
|  | 2 | 8.063 | 0.261 |
|  | 3 | 7.879 | 0.211 |
|  | 4 | 7.924 | 0.197 |
| LSGHippo | 1 | 0.109 | 0.007 |
|  | 2 | 0.105 | 0.007 |
|  | 3 | 0.115 | 0.006 |
|  | 4 | 0.115 | 0.007 |
| LStr | 1 | 2.265 | 0.046 |
|  | 2 | 2.310 | 0.046 |
|  | 3 | 2.270 | 0.057 |
|  | 4 | 2.285 | 0.047 |
| LStriaM | 1 | 0.073 | 0.006 |
|  | 2 | 0.077 | 0.004 |
|  | 3 | 0.081 | 0.006 |
|  | 4 | 0.081 | 0.003 |
| LStriaT | 1 | 0.087 | 0.005 |
|  | 2 | 0.090 | 0.005 |
|  | 3 | 0.092 | 0.006 |
|  | 4 | 0.094 | 0.004 |
| LSubEpendy | 1 | 0.008 | 0.001 |
|  | 2 | 0.008 | 0.001 |
|  | 3 | 0.007 | 0.001 |
|  | 4 | 0.008 | 0.000 |
| LSupCbPed | 1 | 0.099 | 0.003 |
|  | 2 | 0.100 | 0.004 |
|  | 3 | 0.103 | 0.005 |
|  | 4 | 0.100 | 0.007 |
| LSupColl | 1 | 0.850 | 0.026 |
|  | 2 | 0.853 | 0.032 |
|  | 3 | 0.924 | 0.039 |
|  | 4 | 0.910 | 0.033 |
| LSupOli | 1 | 0.096 | 0.008 |
|  | 2 | 0.097 | 0.007 |
|  | 3 | 0.095 | 0.007 |
|  | 4 | 0.097 | 0.008 |
| LThal | 1 | 1.786 | 0.069 |
|  | 2 | 1.829 | 0.070 |
|  | 3 | 1.891 | 0.059 |
|  | 4 | 1.917 | 0.041 |
| RACant | 1 | 0.127 | 0.008 |
|  | 2 | 0.127 | 0.005 |
|  | 3 | 0.124 | 0.007 |
|  | 4 | 0.122 | 0.007 |
| RACpost | 1 | 0.053 | 0.004 |
|  | 2 | 0.050 | 0.002 |
|  | 3 | 0.051 | 0.006 |
|  | 4 | 0.052 | 0.003 |
| RAmy | 1 | 1.660 | 0.064 |
|  | 2 | 1.704 | 0.110 |
|  | 3 | 1.640 | 0.059 |
|  | 4 | 1.631 | 0.100 |
| RAV | 1 | 1.041 | 0.033 |
|  | 2 | 1.038 | 0.032 |
|  | 3 | 1.064 | 0.065 |
|  | 4 | 1.031 | 0.039 |
| RBNST | 1 | 0.142 | 0.006 |
|  | 2 | 0.137 | 0.006 |
|  | 3 | 0.136 | 0.008 |
|  | 4 | 0.140 | 0.005 |
| RBsFB | 1 | 0.518 | 0.023 |
|  | 2 | 0.506 | 0.016 |
|  | 3 | 0.533 | 0.026 |
|  | 4 | 0.526 | 0.032 |
| RCbCtx | 1 | 5.451 | 0.177 |
|  | 2 | 5.424 | 0.182 |
|  | 3 | 5.392 | 0.168 |
|  | 4 | 5.245 | 0.088 |
| RCbrPed | 1 | 0.233 | 0.018 |
|  | 2 | 0.241 | 0.013 |
|  | 3 | 0.250 | 0.011 |
|  | 4 | 0.245 | 0.010 |
| RCC | 1 | 1.919 | 0.072 |
|  | 2 | 1.886 | 0.067 |
|  | 3 | 1.926 | 0.049 |
|  | 4 | 1.923 | 0.063 |
| RCST | 1 | 0.173 | 0.009 |
|  | 2 | 0.175 | 0.009 |
|  | 3 | 0.176 | 0.013 |
|  | 4 | 0.179 | 0.015 |
| RCuneateN | 1 | 0.027 | 0.002 |
|  | 2 | 0.029 | 0.002 |
|  | 3 | 0.030 | 0.004 |
|  | 4 | 0.029 | 0.002 |
| RDG | 1 | 0.401 | 0.023 |
|  | 2 | 0.377 | 0.018 |
|  | 3 | 0.406 | 0.015 |
|  | 4 | 0.397 | 0.025 |
| REnRCx | 1 | 1.318 | 0.042 |
|  | 2 | 1.304 | 0.056 |
|  | 3 | 1.256 | 0.049 |
|  | 4 | 1.268 | 0.066 |
| RFCx | 1 | 5.061 | 0.113 |
|  | 2 | 4.956 | 0.266 |
|  | 3 | 4.859 | 0.130 |
|  | 4 | 4.796 | 0.142 |
| RFacN | 1 | 0.023 | 0.001 |
|  | 2 | 0.023 | 0.001 |
|  | 3 | 0.023 | 0.001 |
|  | 4 | 0.024 | 0.002 |
| RFscRFlx | 1 | 0.030 | 0.002 |
|  | 2 | 0.031 | 0.003 |
|  | 3 | 0.031 | 0.002 |
|  | 4 | 0.030 | 0.001 |
| RFmb | 1 | 0.326 | 0.016 |
|  | 2 | 0.339 | 0.026 |
|  | 3 | 0.350 | 0.020 |
|  | 4 | 0.363 | 0.019 |
| RFStr | 1 | 0.021 | 0.002 |
|  | 2 | 0.020 | 0.003 |
|  | 3 | 0.021 | 0.003 |
|  | 4 | 0.020 | 0.002 |
| RFx | 1 | 0.069 | 0.004 |
|  | 2 | 0.070 | 0.003 |
|  | 3 | 0.071 | 0.004 |
|  | 4 | 0.072 | 0.005 |
| RGP | 1 | 0.321 | 0.026 |
|  | 2 | 0.340 | 0.019 |
|  | 3 | 0.354 | 0.017 |
|  | 4 | 0.355 | 0.021 |
| RHab | 1 | 0.004 | 0.001 |
|  | 2 | 0.004 | 0.001 |
|  | 3 | 0.004 | 0.001 |
|  | 4 | 0.004 | 0.000 |
| RHippo | 1 | 2.316 | 0.061 |
|  | 2 | 2.231 | 0.076 |
|  | 3 | 2.305 | 0.062 |
|  | 4 | 2.290 | 0.102 |
| RvHippo | 1 | 0.2814 | 0.0124 |
|  | 2 | 0.2728 | 0.0134 |
|  | 3 | 0.3108 | 0.0157 |
|  | 4 | 0.3081 | 0.0190 |
| RHypo | 1 | 1.110 | 0.049 |
|  | 2 | 1.111 | 0.043 |
|  | 3 | 1.149 | 0.033 |
|  | 4 | 1.173 | 0.043 |
| RInfCbPed | 1 | 0.081 | 0.007 |
|  | 2 | 0.084 | 0.007 |
|  | 3 | 0.081 | 0.008 |
|  | 4 | 0.085 | 0.007 |
| RInfcoll | 1 | 0.649 | 0.019 |
|  | 2 | 0.651 | 0.020 |
|  | 3 | 0.659 | 0.027 |
|  | 4 | 0.643 | 0.019 |
| RInfOli | 1 | 0.042 | 0.005 |
|  | 2 | 0.042 | 0.003 |
|  | 3 | 0.042 | 0.003 |
|  | 4 | 0.044 | 0.006 |
| RIntC | 1 | 0.257 | 0.013 |
|  | 2 | 0.275 | 0.010 |
|  | 3 | 0.278 | 0.010 |
|  | 4 | 0.285 | 0.018 |
| RLatSpt | 1 | 0.357 | 0.022 |
|  | 2 | 0.342 | 0.017 |
|  | 3 | 0.335 | 0.019 |
|  | 4 | 0.331 | 0.016 |
| RLatVtr | 1 | 0.364 | 0.019 |
|  | 2 | 0.377 | 0.026 |
|  | 3 | 0.355 | 0.020 |
|  | 4 | 0.373 | 0.027 |
| RLatOlfT | 1 | 0.143 | 0.008 |
|  | 2 | 0.149 | 0.010 |
|  | 3 | 0.144 | 0.012 |
|  | 4 | 0.143 | 0.010 |
| RMCbPed | 1 | 0.128 | 0.006 |
|  | 2 | 0.133 | 0.003 |
|  | 3 | 0.130 | 0.006 |
|  | 4 | 0.132 | 0.004 |
| RMamm | 1 | 0.068 | 0.005 |
|  | 2 | 0.072 | 0.006 |
|  | 3 | 0.072 | 0.004 |
|  | 4 | 0.071 | 0.005 |
| RMammTr | 1 | 0.026 | 0.002 |
|  | 2 | 0.026 | 0.002 |
|  | 3 | 0.027 | 0.002 |
|  | 4 | 0.027 | 0.002 |
| RMedSpt | 1 | 0.141 | 0.011 |
|  | 2 | 0.142 | 0.009 |
|  | 3 | 0.137 | 0.011 |
|  | 4 | 0.131 | 0.010 |
| RML | 1 | 0.267 | 0.012 |
|  | 2 | 0.269 | 0.011 |
|  | 3 | 0.275 | 0.014 |
|  | 4 | 0.272 | 0.011 |
| RNac | 1 | 0.438 | 0.022 |
|  | 2 | 0.422 | 0.018 |
|  | 3 | 0.417 | 0.026 |
|  | 4 | 0.412 | 0.018 |
| ROccCx | 1 | 0.770 | 0.038 |
|  | 2 | 0.766 | 0.029 |
|  | 3 | 0.768 | 0.039 |
|  | 4 | 0.747 | 0.032 |
| ROlfBlb | 1 | 2.896 | 0.124 |
|  | 2 | 2.932 | 0.134 |
|  | 3 | 2.857 | 0.098 |
|  | 4 | 2.935 | 0.123 |
| ROptTr | 1 | 0.168 | 0.009 |
|  | 2 | 0.172 | 0.008 |
|  | 3 | 0.177 | 0.009 |
|  | 4 | 0.182 | 0.005 |
| ROlfTub | 1 | 0.403 | 0.021 |
|  | 2 | 0.402 | 0.023 |
|  | 3 | 0.408 | 0.031 |
|  | 4 | 0.386 | 0.026 |
| RPonN | 1 | 0.083 | 0.006 |
|  | 2 | 0.086 | 0.007 |
|  | 3 | 0.088 | 0.008 |
|  | 4 | 0.089 | 0.005 |
| RPreParaSub | 1 | 0.246 | 0.010 |
|  | 2 | 0.225 | 0.017 |
|  | 3 | 0.245 | 0.012 |
|  | 4 | 0.239 | 0.011 |
| RPtempCx | 1 | 9.106 | 0.287 |
|  | 2 | 9.089 | 0.291 |
|  | 3 | 8.981 | 0.158 |
|  | 4 | 8.852 | 0.247 |
| RSGHippo | 1 | 0.092 | 0.007 |
|  | 2 | 0.088 | 0.006 |
|  | 3 | 0.094 | 0.004 |
|  | 4 | 0.094 | 0.006 |
| RStr | 1 | 2.134 | 0.093 |
|  | 2 | 2.168 | 0.087 |
|  | 3 | 2.148 | 0.066 |
|  | 4 | 2.150 | 0.099 |
| RStriaM | 1 | 0.067 | 0.004 |
|  | 2 | 0.070 | 0.004 |
|  | 3 | 0.073 | 0.003 |
|  | 4 | 0.070 | 0.002 |
| RStriaT | 1 | 0.100 | 0.006 |
|  | 2 | 0.105 | 0.007 |
|  | 3 | 0.112 | 0.008 |
|  | 4 | 0.115 | 0.009 |
| RSubEpendy | 1 | 0.006 | 0.001 |
|  | 2 | 0.006 | 0.001 |
|  | 3 | 0.006 | 0.001 |
|  | 4 | 0.006 | 0.001 |
| RSupCbPed | 1 | 0.110 | 0.004 |
|  | 2 | 0.113 | 0.005 |
|  | 3 | 0.118 | 0.010 |
|  | 4 | 0.112 | 0.006 |
| RSupColl | 1 | 0.815 | 0.031 |
|  | 2 | 0.820 | 0.039 |
|  | 3 | 0.873 | 0.036 |
|  | 4 | 0.858 | 0.037 |
| RSupOli | 1 | 0.088 | 0.005 |
|  | 2 | 0.097 | 0.007 |
|  | 3 | 0.091 | 0.009 |
|  | 4 | 0.104 | 0.008 |
| RThal | 1 | 1.725 | 0.051 |
|  | 2 | 1.743 | 0.070 |
|  | 3 | 1.804 | 0.044 |
|  | 4 | 1.835 | 0.057 |

### Detailed structural image analysis


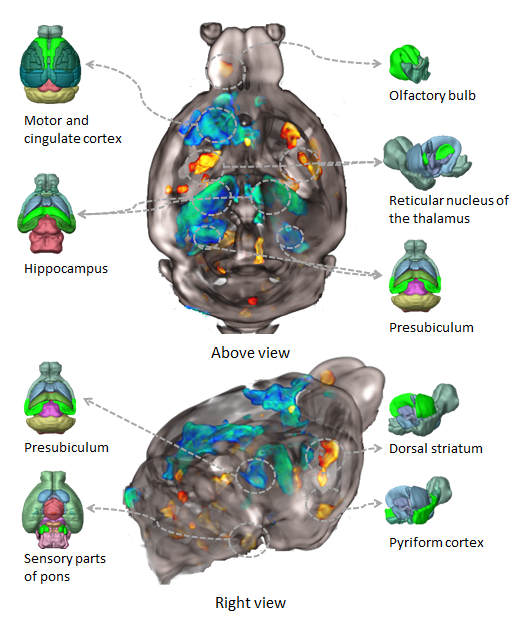


#### Figure S3 Details of structural changes after exposure to chronic intermittent hypoxia in mice with a functional TLR2 gene (TLR2^+/+^).

Image shows statistically significant differences between T*LR2^+/+^CTRL and TLR2^+/+^IH with anatomical models from Brain Explorer 2 for* Allen Mouse Brain Atlas (*http://mouse.brain-map.org/static/atlas*). Most significant enlargements are visible bilaterally in the hippocampi and presubiculi and left motor and cingulate cortices while the reduction of volume is evident bilaterally in the reticular nuclei of the thalamus, right dorsal striatum, right piriform cortex and dorsolateral (sensory) parts of the pons (pontine tegmentum).

**
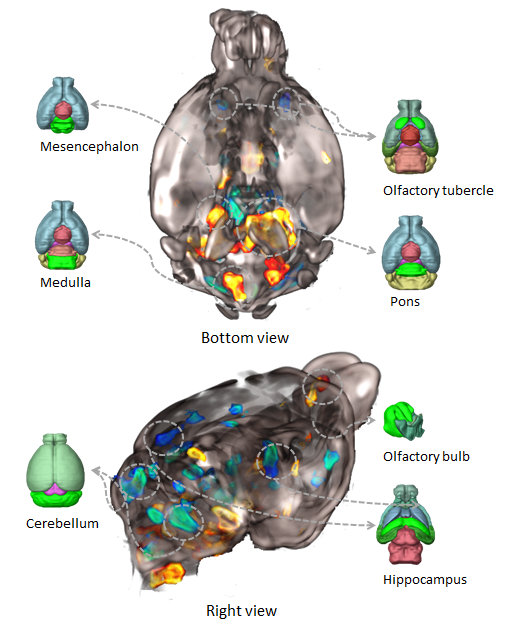
**

#### Figure S4 Details of structural changes after exposure to chronic intermittent hypoxia in mice without a functional TLR2 gene (TLR2^-/-^).

*Image shows statistically significant differences between TLR2^-/-^CTRL and* *TLR2^-/-^IH with anatomical models from Brain Explorer 2 for* Allen Mouse Brain Atlas (*http://mouse.brain-map.org/static/atlas*). Slight increases are visible in the right hippocampus and presubiculum and both olfactory tubercles. More significant enlargements are seen in the parts of the cerebellum while reductions of volume were noted in the pons, mesencephalon and medulla.

### Morphologic and Cellular changes

In our experimental set up, we were unable to demonstrate quantitively gross cellular differences between differential TLR2 genotypes and experimental IH phenotypes to directly account for the primary neuroimaging findings in our study. More specifically, we did not detect gross astroglial proliferation (Figures S4, S5), although a clear protective effect of TLR2 genotype against demyelinating effect of IH experimental protocol was shown (Figure S6).


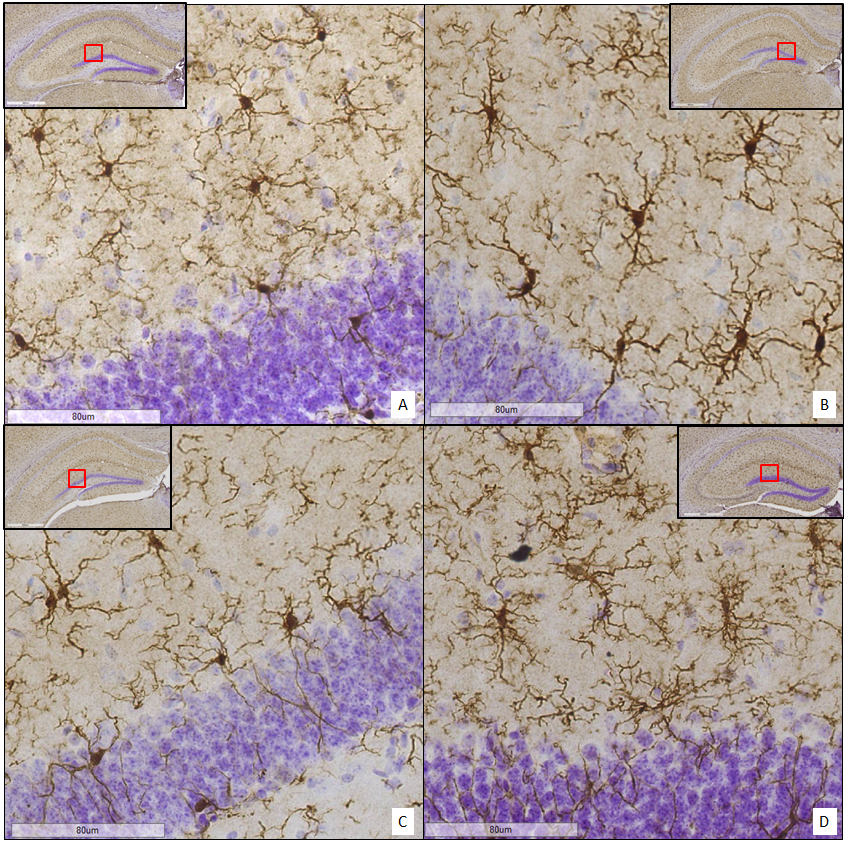


#### Figure S5 Representative images of Iba1 positive cells in the dentate gyrus of four investigated groups.

In the corners of the images are respective areas in red rectangles that the photomicrograph was taken from. Phenotypical distinctions between microglia in four investigated groups are observed, albeit quantatively statistically not found to be significant. A: TLR2^+/+^IH; B: TLR2^+/+^CTRL; C: TLR2^-/-^ IH; D: TLR2^-/-^CTRL; scale bar denotes 80 µm.


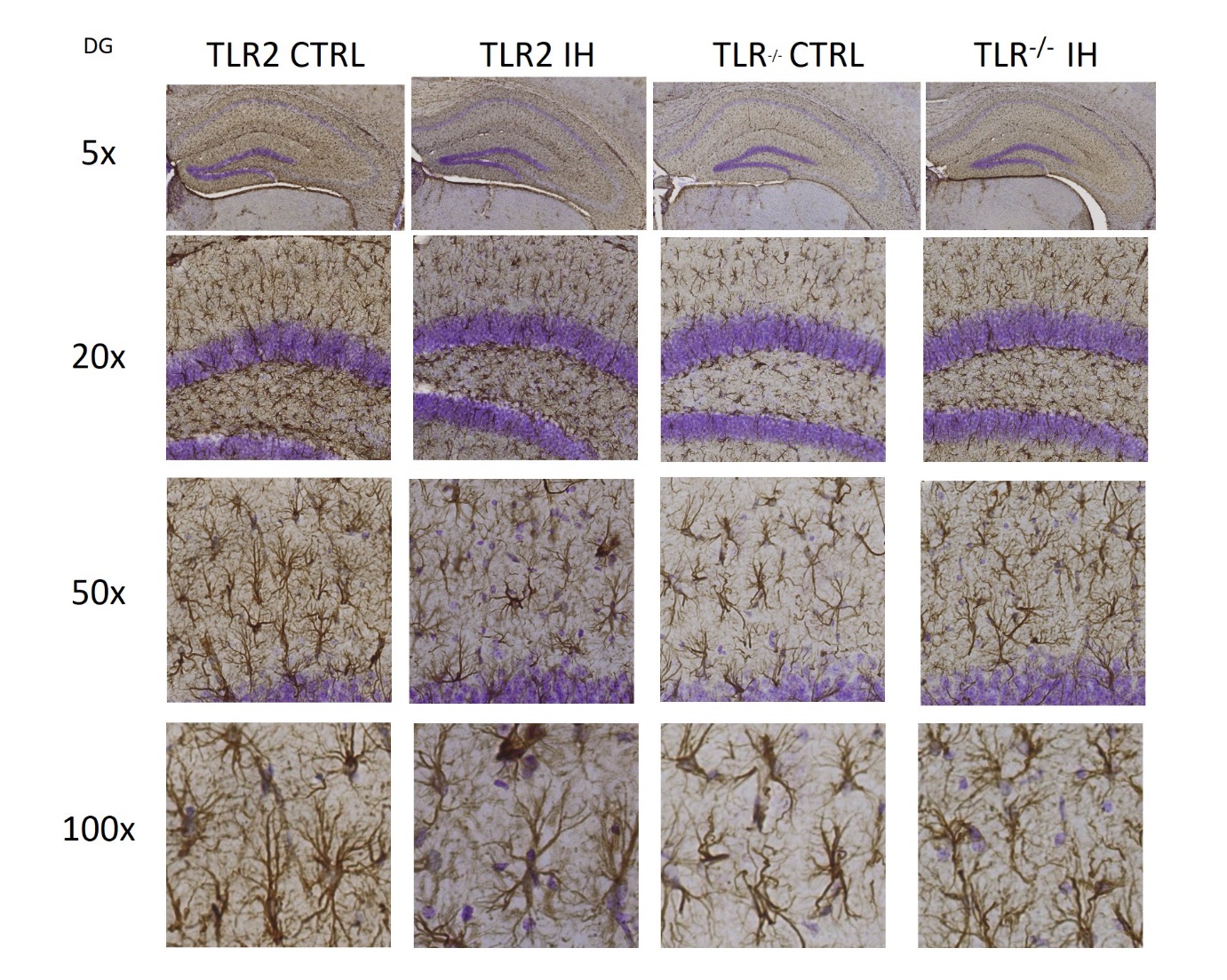


#### Figure S6 Representative images of GFAP positive cells, astrocytes, in the dentate gyrus of hippocampi of four investigated groups under different magnifications.

#### Phenotypical distinctions between astrocytes in four investigated groups are shown, presumably driven by genotype and genotype-driven modulation of response to IH, albeit quantitively statistically not found to be significant. A: TLR2^+/+^IH; B: TLR2^+/+^CTRL; C: TLR2^-/-^ IH; D: TLR2^-/-^CTRL.


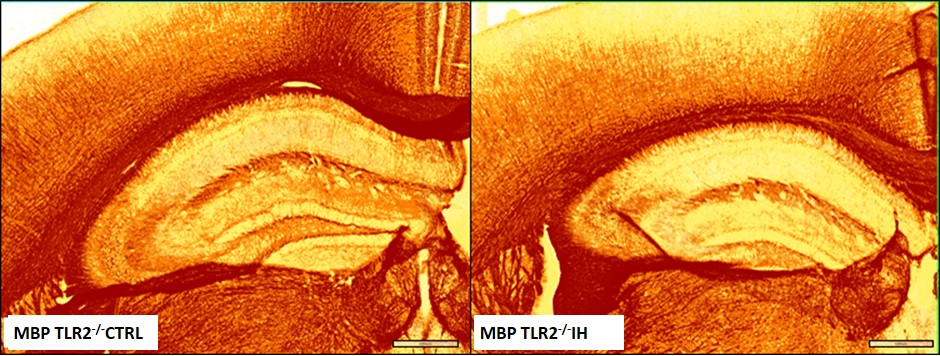


***F******igure S7*** ***Representative images of myelin basic protein staining in the hippocampus.***

Digital thresholding analysis showed the area of MBP positive area to be significantly higher in the TLR2^-/-^ CTRL group (n (mouse) =4; 6 sections) than in the TLR2^-/-^IH group (n =4; 6 sections; F=6.98, *P*=.0286). (5x; *Aperio Image software*; scale bar denotes 500 µm)

*Abbreviations*: MBP- myelin basic protein; IH- intermittent hypoxia; CTRL- control; TLR2^-/-^- TLR2 knock out mouse.


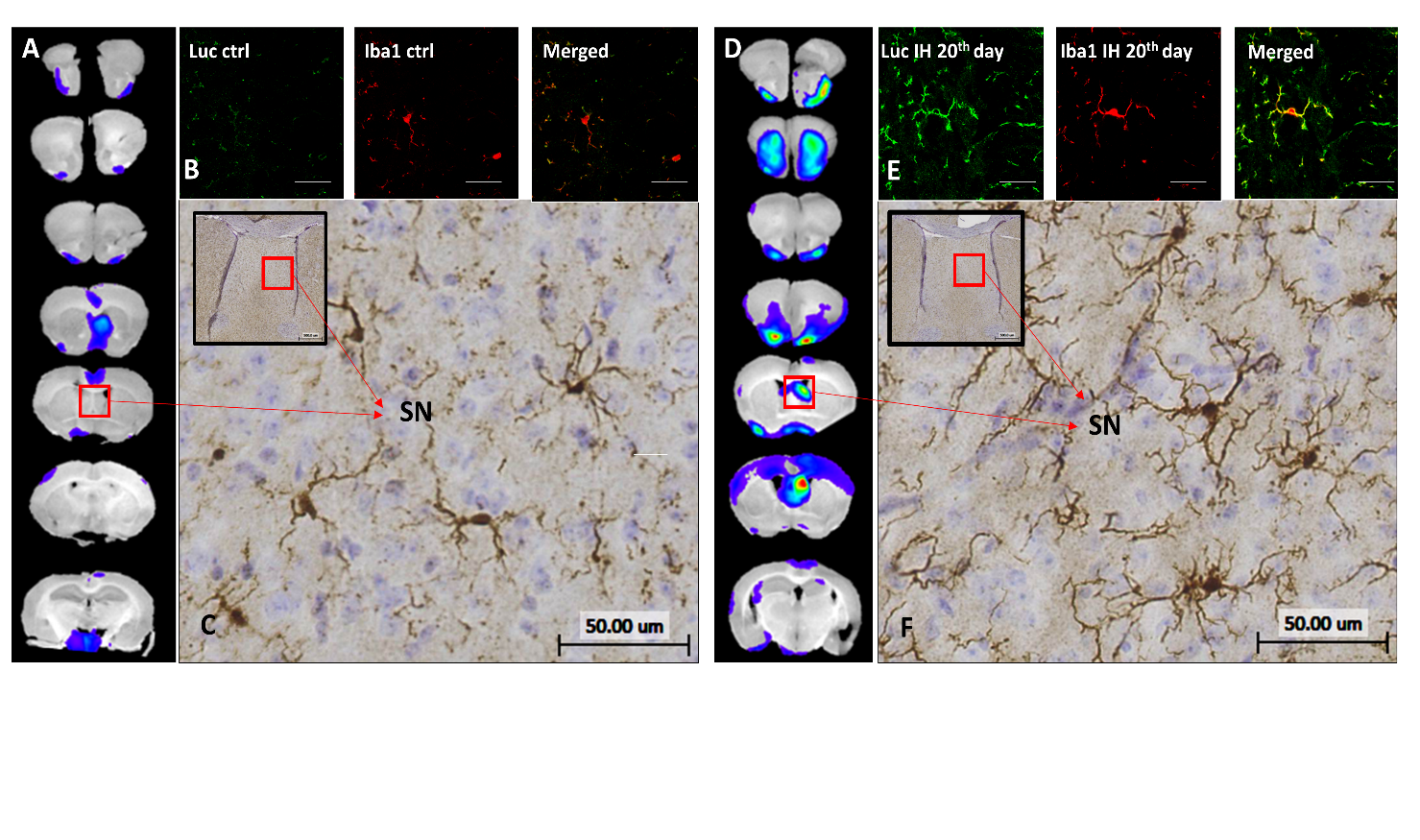


***Figure S8*** ***Ex vivo imaging of bioluminescence signal at 72 hours of experimental protocol co-localises induction of TLR2 microglial signal to basal forebrain and septal nuclei.***

Representative images of control (TLR2^+/+^CTRL; **A-C**) and animals exposed to IH (TLR2^+/+^IH; **D-F**) at 72 hours are shown All animals were exposed to *ex vivo* bioluminescence imaging where TLR2 signal was confirmed in olfactory bulb and anterior olfactory nucleus in both groups, whilst additional signal was noted in the septal nuclei (*red rectangle*) of the IH group only. Immunofluoresecent images of septal region in control (**B**) and IH animals (**E**) demonstrated colocalization of luciferase (*luc*; green) and microglial marker in significant majority of cells (*Iba1*; red; >95%). In **F**, a distinct amoeboid activated morphology of Iba1 positive cells in the septal nuclei region of TLR2^+/+^IH mice is shown. Respective areas in red rectangles depict regions from which photomicrographs were taken. *(X40, Zeiss LSM 510 Meta confocal microscope; scale bar denotes 50 µm.)*

*Abbreviations*: Ctrl- control; TLR2-Toll like receptor 2; IH-intermittent hypoxia, SN- septal nuclei.

A small subset (less than 5%) of luciferase (TLR2^+/+^) positive cells were found to be negative for microglial marker in our study. This is in keeping with several previous studies by our collaborators^1^ that suggested neuronal progenitor origin and/or migrating neuroblasts(e.g. DCX marker positive) for that small subset of cells. In addition, a prominent up-regulation of immediate early gene c-*fos* was also recorded in the hippocampal regions of the cerebral cortex in mice with functional TLR2-system (Table S2).

*Table S2 Quantitative evaluation of several cellular markers in TLR2^+/+^IH (1), TLR^+/+^CTRL (2), TLR2^-/-^IH (3), TLR2^-/-^CTRL (4). The values were normalized as per described protocol.*

*Abbreviations*: cFos- nuclear phosphoprotein ; CA-cornu ammonis; DG – dentate gyrus; GFAP- Glial fibrillary acidic protein ; MBP- myelin basic protein; mean -mean number of cells; Iba1- ionized calcium binding adaptor molecule 1; IH- intermittent hypoxia; CTRL- control; ROI- region of interest; SD – standard deviation; SEM- standard error of mean; TLR2^-/-^ - TLR2 knock out mouse.

| **Cellular marker: ROI** | |  |  |
| --- | --- | --- | --- |
|  |  | **Mean** | **SD** |
| cFos: hippocampus | 1 | 141848.32 | 36115.70 |
|  | 2 | 146464.27 | 27391.78 |
|  | 3 | 98875.72 | 29380.85 |
|  | 4 | 99912.39 | 38871.37 |
| cFos: hypothalamus | 1 | 61836.00 | 7403.99 |
|  | 2 | 58740.55 | 8032.98 |
|  | 3 | 58813.34 | 7003.78 |
|  | 4 | 69054.76 | 25471.26 |
| cFos: thalamus | 1 | 58890.02 | 4700.41 |
|  | 2 | 56962.43 | 4935.93 |
|  | 3 | 54841.82 | 5283.58 |
|  | 4 | 50594.74 | 8370.90 |
| GFAP: hippocampus | 1 | 25.79 | 6.54 |
|  | 2 | 21.50 | 0.76 |
|  | 3 | 23.26 | 11.82 |
|  | 4 | 29.61 | 11.01 |
| GFAP: hypothalamus | 1 | 15.70 | 6.67 |
|  | 2 | 14.15 | 3.64 |
|  | 3 | 14.01 | 9.85 |
|  | 4 | 17.89 | 7.37 |
| GFAP: thalamus | 1 | 3.74 | 2.15 |
|  | 2 | 3.10 | 1.17 |
|  | 3 | 3.09 | 1.19 |
|  | 4 | 5.55 | 2.82 |
| Iba1: thalamus | 1 | 4415.93 | 704.33 |
|  | 2 | 4612.50 | 614.20 |
|  | 3 | 4464.27 | 663.04 |
|  | 4 | 4755.20 | 498.86 |
| Iba1: hypothalamus | 1 | 4306.10 | 1190.58 |
|  | 2 | 4448.58 | 797.97 |
|  | 3 | 4503.18 | 1084.33 |
|  | 4 | 4945.44 | 677.90 |
| Iba1: DG | 1 | 5187.81 | 339.30 |
|  | 2 | 5739.16 | 1111.37 |
|  | 3 | 6094.35 | 783.67 |
|  | 4 | 6046.41 | 471.45 |
| Iba1: CA1 | 1 | 5911.13 | 729.22 |
|  | 2 | 6011.04 | 853.60 |
|  | 3 | 5702.21 | 928.13 |
|  | 4 | 5683.61 | 232.24 |
| Iba1: CA3 | 1 | 5242.53 | 537.55 |
|  | 2 | 5420.07 | 403.26 |
|  | 3 | 5068.17 | 906.29 |
|  | 4 | 5855.42 | 401.24 |
| lba1: dorsal hippocampus | 1 | 5782.44 | 526.51 |
|  | 2 | 5864.36 | 471.21 |
|  | 3 | 5592.27 | 696.95 |
|  | 4 | 5886.82 | 510.65 |
| lba1: ventral hippocampus | 1 | 5058.64 | 511.75 |
|  | 2 | 5389.90 | 537.60 |
|  | 3 | 4986.64 | 651.43 |
|  | 4 | 5085.61 | 310.45 |
| MBP | 1 | 3.45998 | 1.349705 |
|  | 2 | 5.644926 | 2.301069 |
|  | 3 | 5.320819 | 1.030496 |
|  | 4 | 8.049712 | 1.789514 |

#### Table S3. Average neuroplastin immunoreactivity values as integrated optical density (IOD) are shown for all major hippocampal regions.

*Abbreviations*: DG – dentate gyrus. CTRL – control; CA – Ammon’s horn (*Cornu Ammonis*), IH- intermittent hypoxia, SD- standard deviation, str. – stratum, TLR2^-/-^ - TLR2 knock out mouse.

|  | **REGION** | **TLR2^+/+^IH** | | **TLR2^+/+^CTRL** | | **TLR2^-/-^ IH** | | **TLR2^-/-^ CTRL** | |
| --- | --- | --- | --- | --- | --- | --- | --- | --- | --- |
|  |  | mean | SD | mean | SD | mean | SD | mean | SD |
| DG | *str. granulare* | 1551837.806 | 92460.08074 | 1625417.514 | 280262.4315 | 1473234.185 | 220753.828 | 1188363.033 | 78114.9897 |
|  | *str. moleculare* | 1357669.236 | 55887.72664 | 1421728.306 | 264159.4369 | 1296908.065 | 147027.1163 | 1082671.422 | 49815.32292 |
| CA3 | *str. pyramidale* | 1480109.458 | 117896.6354 | 1564299.403 | 192327.5423 | 1339265.222 | 152809.9737 | 1145629.033 | 167876.599 |
|  | *str. radiatum* | 1426939.75 | 123935.5783 | 1484091.972 | 174778.397 | 1293531.472 | 140770.0314 | 1133350.922 | 133030.3292 |
|  | *str. oriens* | 1417715.167 | 202002.4107 | 1538039.806 | 160727.3422 | 1288390.306 | 156226.1885 | 1128826.322 | 118559.0546 |
| CA2 | *str. pyramidale* | 1450676.014 | 205226.6957 | 1563036.736 | 199066.5075 | 1363181.694 | 140448.6548 | 1112153.056 | 166972.7896 |
|  | *str. radiatum* | 1375802.9 | 109941.6369 | 1470989.83 | 207154.8051 | 1303567.11 | 150504.0415 | 1118247.89 | 144971.1249 |
|  | *str. oriens* | 1419759.792 | 204980.1828 | 1518213.833 | 172862.7509 | 1335268.722 | 162498.5641 | 1101295.156 | 126852.9329 |
| CA1 | *str. pyramidale* | 1634771.259 | 128044.1863 | 1526307.852 | 178344.4649 | 1444880.037 | 233533.1714 | 1233602.500 | 130180.1678 |
|  | *str. radiatum* | 1481651.407 | 139739.1881 | 1419492.648 | 137166.17 | 1353984.713 | 217919.114 | 1175047.389 | 66198.10435 |
|  | *str. oriens* | 1522651.352 | 74172.47761 | 1483661.815 | 109709.5437 | 1366674.028 | 220241.6968 | 1182495.722 | 144191.7691 |


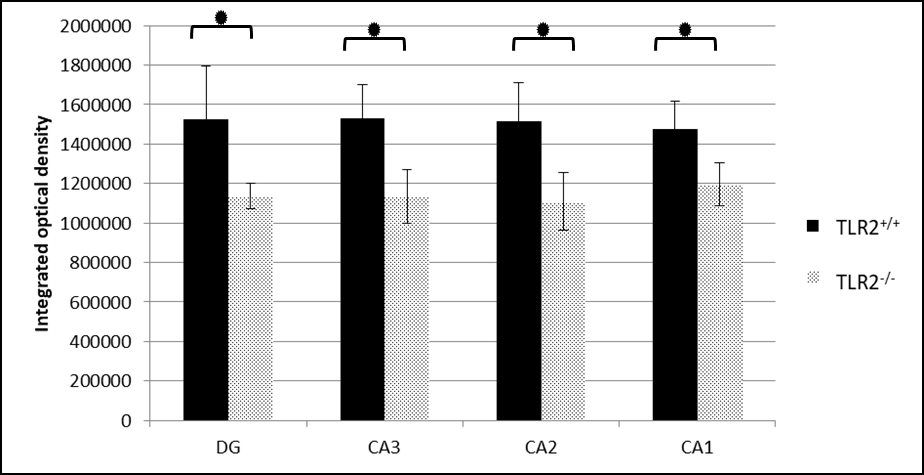


**A**


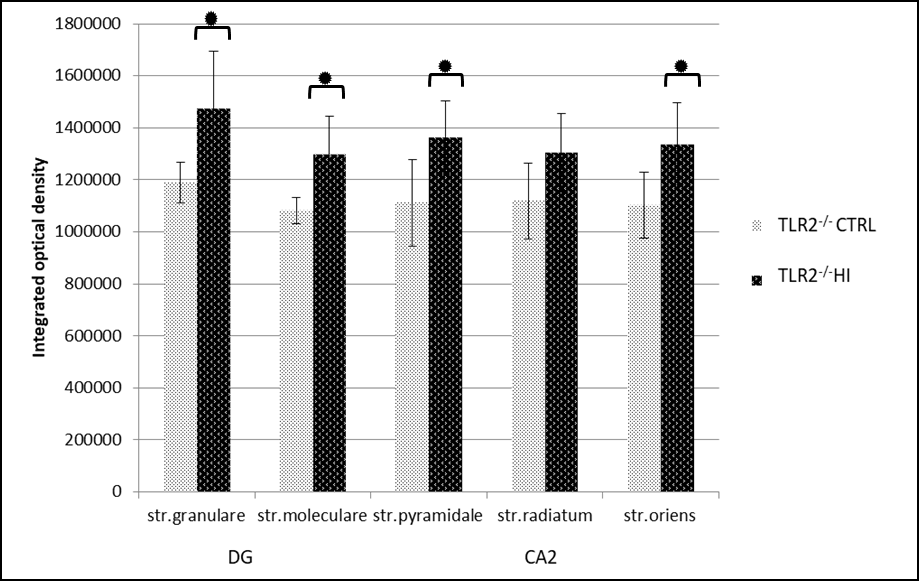


**B**

***Figure S9. Significantly higher neuroplastin immunoreactivity is demonstrated in animals with functional TLR2 systems (TLR2^+/+^) in all major hippocampal regions (A).***  **However, a significant increase of neuroplastin immunoreactivity following IH protocol was only recorded in TLR2^-/-^ animals (B) in *dentate gyrus* (DG), *stratum granulare* (t=-2.72;df=9; *P* =.023) and *stratum moleculare* (t=-3.09;df=9; *P* =.013), and in CA2 region *stratum pyramidale* (t=-2.71; df=9; *P*=.024) and *stratum oriens* (t=-2.61;df=9;*P*=.028)**. Error bars denote standard deviation (SD). * equals *P* < .05, Student t-test.

*Abbreviations*: CA- Cornu Ammonis; CTRL – control conditions; DG – dentate gyrus; H-hippocampus; IH-intermittent hypoxia protocol, str.- stratum; TLR2-Toll like receptor 2.

#### Integration of Structural Neuroimaging and mRNA Brain Expression Maps

#### Table S4. Linear regression results are shown. Green field denote statistical significance. Abbreviations: ARHGEF6- Rho Guanine Nucleotide Exchange Factor 6; BDNF- the brain derived neurotrophic factor; CAMK2A- Calcium/calmodulin dependent protein kinase II alpha; CCK- Cholecystokinin; PCP4; MOBP- Myelin Associated Oligodendrocyte Basic Protein; ; NPTN-neuroplastin FN1- Fibronectin 1; HOMER1; RASGRP1- RAS guanyl nucleotide-releasing protein 1; TLR2^-/-^ - TLR2 knock out mouse; TLR2^+/+^(functional TLR2).

| **MR Parameter (group)** | **Variable** | **PCP4** | **MOBP** | **FN1** | **ARHGEF6** | **CAMK2A** | **HOMER1** | **CCK** | **BDNF** | **RASGRP1** | **NPTN** |
| --- | --- | --- | --- | --- | --- | --- | --- | --- | --- | --- | --- |
| V (TLR2^+/+^) | Pearson Correlation | .103 | -.467 | .635* | .214 | .644* | .491 | .418 | .534 | .580* | .532 |
|  | Sig. (2-tailed) | .751 | .126 | .027 | .504 | .024 | .105 | .201 | .074 | .048 | .075 |
|  | N | 12 | 12 | 12 | 12 | 12 | 12 | 11 | 12 | 12 | 12 |
| V (TLR2 ^-/-^) | Pearson Correlation | .220 | -.297 | .162 | .047 | .376 | .315 | .088 | .132 | .404 | .419 |
|  | Sig. (2-tailed) | .492 | .348 | .615 | .884 | .229 | .318 | .796 | .683 | .193 | .175 |
|  | N | 12 | 12 | 12 | 12 | 12 | 12 | 11 | 12 | 12 | 12 |
| T1 (TLR2^+/+^) | Pearson Correlation | .194 | -.468 | .682* | .685* | .650* | .489 | .797** | .813** | .613* | .566 |
|  | Sig. (2-tailed) | .546 | .125 | .015 | .014 | .022 | .107 | .003 | .001 | .034 | .055 |
|  | N | 12 | 12 | 12 | 12 | 12 | 12 | 11 | 12 | 12 | 12 |
| T1(TLR2 ^-/-^) | Pearson Correlation | .157 | -.160 | .505 | .196 | .300 | .104 | .557 | .450 | .253 | .165 |
|  | Sig. (2-tailed) | .626 | .618 | .094 | .541 | .343 | .747 | .075 | .142 | .427 | .608 |
|  | N | 12 | 12 | 12 | 12 | 12 | 12 | 11 | 12 | 12 | 12 |
| T2 (TLR2^+/+^) | Pearson Correlation | -.454 | .389 | -.215 | -.332 | -.691* | -.469 | -.454 | -.457 | -.700* | -.559 |
|  | Sig. (2-tailed) | .138 | .211 | .501 | .292 | .013 | .124 | .160 | .136 | .011 | .059 |
|  | N | 12 | 12 | 12 | 12 | 12 | 12 | 11 | 12 | 12 | 12 |
| T2 (TLR2^-/-^) | Pearson Correlation | -.515 | -.002 | .016 | .034 | -.549 | -.237 | -.268 | -.297 | -.522 | -.474 |
|  | Sig. (2-tailed) | .087 | .994 | .961 | .918 | .065 | .457 | .425 | .349 | .082 | .120 |
|  | N | 12 | 12 | 12 | 12 | 12 | 12 | 11 | 12 | 12 | 12 |

* refers to statistically significant results (*P* <.05)

#### Table S5. The list of major neuroplasticity genes that were investigated.

| Group | Genes | Relevant References |
| --- | --- | --- |
| Sleep homeostasis (Neuroplasticity) | BDNF, FOS, FOSB, FOSL1, FOSL2, PAX6, AVPR1B, CCK, TLR2, HOMER1, ARC, EGR1, GSK3B, CAMK2A, GAP43, NGF, DISC1, COMT | ^27, 28, 29, 30, 31, 32, 33^ ^34^ ^26^ ^35^ ^36^ ^37^ ^38^ ^39^ ^40, 41^ |
| Neuroplasticity | FIGF, NGFR, NRG1, GSK3A, CREB1, CREB3, CRTC1, GPM6A, NCAM1, NCAM2, REST, RGS14, PCP4, ARHGEF6, DRD3, ITM2B, LRRTM2, MDK, RASGRP1, SLITRK5, SSTR4, NPTN, EPHA2, EMP1, FN1, HES5, SOX11, CORO1A, NR2E1, GNAQ, CLK1, USF2, SRF | ^42, 43, 44, 45, 46, 47, 48, 49, 50, 51, 52, 53, 54, 55, 56, 57, 58, 59, 60, 61, 62, 63, 64, 65, 66, 67^ ^68, 69, 70, 71, 72^ |
|  |  |  |
| Growth/development | PROX1, SOX2, MSX1 | ^73, 74, 75, 76, 77, 78^ |
| Myelin formation | CLDN11, MOBP | ^79^, ^80^, ^81^, ^82^ |
| Immunoactive factor | CAMP | ^83^, ^84^, ^85^, ^86^ |
| Angiogenic factor | ANGPT22 | ^87^, ^88^,  ^89^, ^90^, ^91^ |

## Behavioural Changes

#### Table S6. Values of behavioural test parameters in four groups of animals: TLR2^+/+^IH (1; n=15), TLR^+/+^CTRL (2; n=12), TLR2^-/-^IH (3; n=16), TLR2^-/-^CTRL (4; n=8).

*Abbreviations*: OF- open field; SD- standard deviation; TST- tail suspension test.

| **Behavioural test: test parameter** | |  |  |  |
| --- | --- | --- | --- | --- |
|  |  | **Mean** | *SD* | *SEM* |
| OF: Duration | 1 | 599.99 | 0.03 | 0.01 |
|  | 2 | 600.00 | 0.00 | 0.00 |
|  | 3 | 600.00 | 0.00 | 0.00 |
|  | 4 | 600.00 | 0.00 | 0.00 |
| OF: Distance | 1 | 28.85 | 5.81 | 1.68 |
|  | 2 | 24.93 | 9.93 | 3.31 |
|  | 3 | 28.11 | 4.67 | 1.21 |
|  | 4 | 29.18 | 10.12 | 3.58 |
| OF: Mean speed | 1 | 0.05 | 0.01 | 0.00 |
|  | 2 | 0.05 | 0.01 | 0.00 |
|  | 3 | 0.05 | 0.01 | 0.00 |
|  | 4 | 0.05 | 0.02 | 0.01 |
| OF: Time mobile | 1 | 486.39 | 43.37 | 12.52 |
|  | 2 | 458.16 | 45.94 | 16.24 |
|  | 3 | 450.40 | 48.80 | 12.60 |
|  | 4 | 421.79 | 64.09 | 22.66 |
| OF: Time immobile | 1 | 113.59 | 43.35 | 12.51 |
|  | 2 | 141.84 | 45.94 | 16.24 |
|  | 3 | 149.60 | 48.80 | 12.60 |
|  | 4 | 178.21 | 64.09 | 22.66 |
| OF: Mobile episodes | 1 | 27.17 | 7.47 | 2.16 |
|  | 2 | 32.25 | 8.36 | 2.96 |
|  | 3 | 35.20 | 8.69 | 2.24 |
|  | 4 | 34.88 | 7.77 | 2.75 |
| OF: Immobile episodes | 1 | 26.67 | 7.67 | 2.21 |
|  | 2 | 32.00 | 8.30 | 2.93 |
|  | 3 | 34.73 | 8.71 | 2.25 |
|  | 4 | 34.13 | 7.68 | 2.72 |
| OF: Line crossings | 1 | 165.58 | 51.73 | 14.93 |
|  | 2 | 139.75 | 38.32 | 13.55 |
|  | 3 | 167.47 | 37.84 | 9.77 |
|  | 4 | 146.38 | 37.18 | 13.14 |
| OF: Absolute turn angle | 1 | 56252.17 | 11455.53 | 3306.93 |
|  | 2 | 43144.38 | 8385.90 | 2964.86 |
|  | 3 | 41261.33 | 8279.64 | 2137.79 |
|  | 4 | 36473.63 | 8784.88 | 3105.92 |
| OF: Max speed | 1 | 0.03 | 0.00 | 0.00 |
|  | 2 | 0.03 | 0.01 | 0.00 |
|  | 3 | 0.03 | 0.00 | 0.00 |
|  | 4 | 0.04 | 0.01 | 0.00 |
| OF: Rotation | 1 | 31.00 | 6.62 | 1.91 |
|  | 2 | 26.38 | 4.44 | 1.57 |
|  | 3 | 34.33 | 7.11 | 1.84 |
|  | 4 | 33.75 | 9.50 | 3.36 |
| OF: Clockwise rotations | 1 | 16.58 | 4.80 | 1.38 |
|  | 2 | 13.25 | 6.69 | 2.37 |
|  | 3 | 16.27 | 6.53 | 1.69 |
|  | 4 | 14.13 | 4.49 | 1.59 |
| OF: Anti-clockwise rotations | 1 | 14.42 | 5.33 | 1.54 |
|  | 2 | 13.13 | 2.75 | 0.97 |
|  | 3 | 18.07 | 7.21 | 1.86 |
|  | 4 | 19.63 | 7.71 | 2.73 |
| YM: Distance | 1 | 48.05 | 9.71 | 2.80 |
|  | 2 | 46.45 | 8.18 | 2.47 |
|  | 3 | 50.62 | 5.75 | 1.66 |
|  | 4 | 42.64 | 4.69 | 1.77 |
| YM: Mean speed | 1 | 0.08 | 0.02 | 0.00 |
|  | 2 | 0.08 | 0.01 | 0.00 |
|  | 3 | 0.08 | 0.01 | 0.00 |
|  | 4 | 0.07 | 0.01 | 0.00 |
| YM: Time mobile | 1 | 440.25 | 42.47 | 12.26 |
|  | 2 | 424.42 | 27.66 | 8.34 |
|  | 3 | 455.93 | 29.42 | 8.49 |
|  | 4 | 413.09 | 13.93 | 5.26 |
| YM: Time immobile | 1 | 159.75 | 42.47 | 12.26 |
|  | 2 | 175.58 | 27.66 | 8.34 |
|  | 3 | 144.08 | 29.42 | 8.49 |
|  | 4 | 186.91 | 13.93 | 5.26 |
| YM: Mobile episodes | 1 | 37.58 | 5.28 | 1.52 |
|  | 2 | 44.36 | 5.75 | 1.73 |
|  | 3 | 34.33 | 4.68 | 1.35 |
|  | 4 | 38.86 | 7.22 | 2.73 |
| YM: Immobile episodes | 1 | 36.50 | 5.32 | 1.53 |
|  | 2 | 44.09 | 5.75 | 1.73 |
|  | 3 | 33.83 | 4.65 | 1.34 |
|  | 4 | 38.29 | 7.13 | 2.70 |
| YM: Line crossings | 1 | 141.08 | 22.47 | 6.49 |
|  | 2 | 140.45 | 28.39 | 8.56 |
|  | 3 | 163.50 | 19.30 | 5.57 |
|  | 4 | 163.14 | 37.62 | 14.22 |
| YM: Absolute turn angle | 1 | 46991.00 | 10471.86 | 3022.97 |
|  | 2 | 43429.45 | 6819.24 | 2056.08 |
|  | 3 | 42262.33 | 7423.61 | 2143.01 |
|  | 4 | 50424.00 | 8257.70 | 3121.12 |
| YM: Max speed | 1 | 0.91 | 0.50 | 0.14 |
|  | 2 | 0.82 | 0.45 | 0.13 |
|  | 3 | 0.57 | 0.06 | 0.02 |
|  | 4 | 0.53 | 0.08 | 0.03 |
| YM: Rotations | 1 | 20.42 | 4.06 | 1.17 |
|  | 2 | 21.64 | 4.65 | 1.40 |
|  | 3 | 26.75 | 3.17 | 0.91 |
|  | 4 | 25.57 | 2.07 | 0.78 |
| YM: Clockwise rotations | 1 | 12.83 | 5.04 | 1.46 |
|  | 2 | 12.64 | 5.39 | 1.63 |
|  | 3 | 15.33 | 3.98 | 1.15 |
|  | 4 | 11.00 | 3.87 | 1.46 |
| YM: Anti-clockwise rotations | 1 | 7.58 | 3.78 | 1.09 |
|  | 2 | 9.00 | 5.04 | 1.52 |
|  | 3 | 11.42 | 4.80 | 1.38 |
|  | 4 | 14.57 | 4.43 | 1.67 |
| YM: Path efficiency (%) | 1 | 1.05 | 0.58 | 0.16 |
|  | 2 | 1.09 | 0.66 | 0.20 |
|  | 3 | 0.625 | 0.49 | 0.14 |
|  | 4 | 1.04 | 0.53 | 0.20 |
| YM: Spontaneous alternation % | 1 | 63.24 | 5.65 | 1.63 |
|  | 2 | 61.03 | 9.48 | 2.86 |
|  | 3 | 59.65 | 6.02 | 1.74 |
|  | 4 | 58.55 | 4.36 | 1.65 |
| TST: Duration | 1 | 360.00 | 0.00 | 0.00 |
|  | 2 | 360.00 | 0.00 | 0.00 |
|  | 3 | 360.00 | 0.00 | 0.00 |
|  | 4 | 358.81 | 3.14 | 1.19 |
| TST: number of presses | 1 | 33.86 | 11.94 | 3.19 |
|  | 2 | 31.33 | 10.17 | 3.39 |
|  | 3 | 28.33 | 9.56 | 2.76 |
|  | 4 | 36.00 | 8.16 | 3.09 |
| TST: Mirovanje : time pressed | 1 | 103.72 | 48.09 | 12.85 |
|  | 2 | 152.68 | 34.69 | 11.56 |
|  | 3 | 99.43 | 33.17 | 9.57 |
|  | 4 | 144.90 | 56.08 | 21.20 |
| TST: latency 1st press | 1 | 27.65 | 7.11 | 1.90 |
|  | 2 | 27.37 | 12.79 | 4.26 |
|  | 3 | 45.04 | 22.08 | 6.37 |
|  | 4 | 30.14 | 12.38 | 4.68 |
| TST: latency 1st release | 1 | 28.15 | 7.44 | 1.99 |
|  | 2 | 27.81 | 13.16 | 4.39 |
|  | 3 | 45.42 | 22.18 | 6.40 |
|  | 4 | 30.99 | 11.84 | 4.48 |
| TST: longest press | 1 | 18.28 | 10.79 | 2.88 |
|  | 2 | 23.10 | 7.84 | 2.61 |
|  | 3 | 17.87 | 6.89 | 1.99 |
|  | 4 | 17.80 | 6.76 | 2.55 |
| TST: shortest press | 1 | 0.11 | 0.09 | 0.02 |
|  | 2 | 0.12 | 0.08 | 0.03 |
|  | 3 | 0.11 | 0.07 | 0.02 |
|  | 4 | 0.10 | 0.06 | 0.02 |
| TST: mean press duration | 1 | 3.30 | 1.82 | 0.49 |
|  | 2 | 5.16 | 1.53 | 0.51 |
|  | 3 | 3.53 | 1.13 | 0.33 |
|  | 4 | 4.10 | 1.79 | 0.68 |
| TST: press frequency | 1 | 0.09 | 0.03 | 0.01 |
|  | 2 | 0.09 | 0.03 | 0.01 |
|  | 3 | 0.08 | 0.03 | 0.01 |
|  | 4 | 0.10 | 0.02 | 0.01 |

## 6.Weight Changes

#### Table S7. Timeline of changes in weight is shown in four investigated groups. Values are shown as percentage of change from the baseline weight ± standard deviation. n denotes number of animals per group; animals were grouped according to their genotype and intervention. Groups: 1 = TLR2^+/+^IH; 2= TLR2^+/+^CTRL; 3= TLR2^-/-^ IH; 4= TLR2^-/-^ CTRL; Abbreviations: SD- standard deviation.

| **∆ Weight (%)** | | **Mean** | **SD** |
| --- | --- | --- | --- |
|  |  |  |  |
| Third day *vs.* baseline | 1 | -6.27 | 5.39 |
|  | 2 | 1.17 | 2.86 |
|  | 3 | -4.75 | 2.32 |
|  | 4 | -1.50 | 1.20 |
| Sixth day *vs* baseline | 1 | -4.53 | 4.94 |
|  | 2 | 3.58 | 2.39 |
|  | 3 | -3.31 | 2.36 |
|  | 4 | 0.63 | 1.41 |
| Ninth day *vs* baseline | 1 | -4.27 | 5.13 |
|  | 2 | 6.25 | 10.14 |
|  | 3 | -3.81 | 2.17 |
|  | 4 | 0.00 | 1.31 |
| Weight difference 21^st^ day *vs.* baseline | 1 | -2.00 | 6.19 |
|  | 2 | 5.60 | 4.34 |
|  | 3 | -4.89 | 3.76 |
|  | 4 | 0.33 | 1.03 |

#### Table S8. Significance of differences in weight changes during exposure to intermittent hypoxia versus those in their respective controls is shown for groups with functional (TLR2^+/+^) and non-functional gene (TLR2^-/-^). One-way ANOVA with Bonferroni post-hoc correction for multiple comparison presented in S7.

| **∆ Weight** | **TLR2^+/+^** | | **TLR2^-/-^** | |
| --- | --- | --- | --- | --- |
|  | F values | *P* values | F values | *P* values |
| Third day vs. baseline | 18.52 | <.001 | 1.40 | .234 |
| Sixth day vs baseline | 26.14 | <.001 | 5.50 | .038 |
| Ninth day vs. baseline | 12.37 | <.001 | 0.12 | .797 |
| 21^st^ day vs. baseline | 5.58 | .036 | 2.73 | .158 |

***References:***

1. Lalancette-Hebert M, Phaneuf D, Soucy G, Weng YC, Kriz J. Live imaging of Toll-like receptor 2 response in cerebral ischaemia reveals a role of olfactory bulb microglia as modulators of inflammation. *Brain* **132**, 940-954 (2009).

2. Pexels PbAFf. Brown and White Mice. (ed^(eds). Pexels (2019).

3. Abelaira HM, Reus GZ, Quevedo J. Animal models as tools to study the pathophysiology of depression. *Braz J Psychiatry* **35 Suppl 2**, S112-120 (2013).

4. Cahill LS*, et al.* MRI-detectable changes in mouse brain structure induced by voluntary exercise. *Neuroimage* **113**, 175-183 (2015).

5. West MJ, Slomianka L, Gundersen HJ. Unbiased stereological estimation of the total number of neurons in thesubdivisions of the rat hippocampus using the optical fractionator. *Anat Rec* **231**, 482-497 (1991).

6. Tschanz SA, Burri PH, Weibel ER. A simple tool for stereological assessment of digital images: the STEPanizer. *J Microsc* **243**, 47-59 (2011).

7. Mandarim-de-Lacerda CA, Del Sol, M. Tips for Studies with Quantitative Morphology (Morphometry and Stereology). *Int J Morphol* **35**, 1482–1494 (2017).

8. Westphal R*, et al.* Characterization of gray matter atrophy following 6-hydroxydopamine lesion of the nigrostriatal system. *Neuroscience* **334**, 166-179 (2016).

9. Abbink MR*, et al.* Characterization of astrocytes throughout life in wildtype and APP/PS1 mice after early-life stress exposure. *J Neuroinflammation* **17**, 91 (2020).

10. Khodanovich M*, et al.* Quantitative Imaging of White and Gray Matter Remyelination in the Cuprizone Demyelination Model Using the Macromolecular Proton Fraction. *Cells* **8**, (2019).

11. Park SJ, Lee JY, Kim SJ, Choi SY, Yune TY, Ryu JH. Toll-like receptor-2 deficiency induces schizophrenia-like behaviors in mice. *Sci Rep* **5**, 8502 (2015).

12. Rafa-Zablocka K, Kreiner G, Baginska M, Kusmierczyk J, Parlato R, Nalepa I. Transgenic mice lacking CREB and CREM in noradrenergic and serotonergic neurons respond differently to common antidepressants on tail suspension test. *Sci Rep* **7**, 13515 (2017).

13. Deoni SC. Transverse relaxation time (T2) mapping in the brain with off-resonance correction using phase-cycled steady-state free precession imaging. *J Magn Reson Imaging* **30**, 411-417 (2009).

14. Wood TC. Improved formulas for the two optimum VFA flip-angles. *Magn Reson Med* **74**, 1-3 (2015).

15. Yarnykh VL. Optimal radiofrequency and gradient spoiling for improved accuracy of T1 and B1 measurements using fast steady-state techniques. *Magn Reson Med* **63**, 1610-1626 (2010).

16. Yarnykh VL. Actual flip-angle imaging in the pulsed steady state: a method for rapid three-dimensional mapping of the transmitted radiofrequency field. *Magn Reson Med* **57**, 192-200 (2007).

17. Jenkinson M, Beckmann CF, Behrens TE, Woolrich MW, Smith SM. Fsl. *Neuroimage* **62**, 782-790 (2012).

18. Avants BB, Tustison NJ, Song G, Cook PA, Klein A, Gee JC. A reproducible evaluation of ANTs similarity metric performance in brain image registration. *Neuroimage* **54**, 2033-2044 (2011).

19. Wood TC. QUIT: QUantitative Imaging Tools.

20. Wood TC*, et al.* Whole-brain ex-vivo quantitative MRI of the cuprizone mouse model. *PeerJ* **4**, e2632 (2016).

21. Dorr AE, Lerch JP, Spring S, Kabani N, Henkelman RM. High resolution three-dimensional brain atlas using an average magnetic resonance image of 40 adult C57Bl/6J mice. *Neuroimage* **42**, 60-69 (2008).

22. Smith SM, Nichols TE. Threshold-free cluster enhancement: addressing problems of smoothing, threshold dependence and localisation in cluster inference. *Neuroimage* **44**, 83-98 (2009).

23. Winkler AM, Ridgway GR, Webster MA, Smith SM, Nichols TE. Permutation inference for the general linear model. *Neuroimage* **92**, 381-397 (2014).

24. Allen EA, Erhardt EB, Calhoun VD. Data visualization in the neurosciences: overcoming the curse of dimensionality. *Neuron* **74**, 603-608 (2012).

25. Herrera-Molina R*, et al.* Neuroplastin deletion in glutamatergic neurons impairs selective brain functions and calcium regulation: implication for cognitive deterioration. *Sci Rep* **7**, 7273 (2017).

26. Basheer R, Brown R, Ramesh V, Begum S, McCarley RW. Sleep deprivation-induced protein changes in basal forebrain: implications for synaptic plasticity. *Journal of neuroscience research* **82**, 650-658 (2005).

27. Schmitt K, Holsboer-Trachsler E, Eckert A. BDNF in sleep, insomnia, and sleep deprivation. *Annals of medicine* **48**, 42-51 (2016).

28. Shiromani PJ, Basheer R, Thakkar J, Wagner D, Greco MA, Charness ME. Sleep and wakefulness in c-fos and fos B gene knockout mice. *Brain research Molecular brain research* **80**, 75-87 (2000).

29. Terao A, Greco MA, Davis RW, Heller HC, Kilduff TS. Region-specific changes in immediate early gene expression in response to sleep deprivation and recovery sleep in the mouse brain. *Neuroscience* **120**, 1115-1124 (2003).

30. Hanish AE, Butman JA, Thomas F, Yao J, Han JC. Pineal hypoplasia, reduced melatonin and sleep disturbance in patients with PAX6 haploinsufficiency. *Journal of sleep research*, (2015).

31. van West D*, et al.* A major SNP haplotype of the arginine vasopressin 1B receptor protects against recurrent major depression. *Molecular psychiatry* **9**, 287-292 (2004).

32. Sei M, Sei H, Shima K. Spontaneous activity, sleep, and body temperature in rats lacking the CCK-A receptor. *Physiology & behavior* **68**, 25-29 (1999).

33. Sartorius T*, et al.* Toll-like receptors 2 and 4 impair insulin-mediated brain activity by interleukin-6 and osteopontin and alter sleep architecture. *FASEB journal : official publication of the Federation of American Societies for Experimental Biology* **26**, 1799-1809 (2012).

34. Ramos OV, Torterolo P, Lim V, Chase MH, Sampogna S, Yamuy J. The role of mesopontine NGF in sleep and wakefulness. *Brain research* **1413**, 9-23 (2011).

35. Wisor JP. A metabolic-transcriptional network links sleep and cellular energetics in the brain. *Pflugers Archiv : European journal of physiology* **463**, 15-22 (2012).

36. Ribeiro S, Goyal V, Mello CV, Pavlides C. Brain gene expression during REM sleep depends on prior waking experience. *Learning & memory* **6**, 500-508 (1999).

37. Gronli J, Soule J, Bramham CR. Sleep and protein synthesis-dependent synaptic plasticity: impacts of sleep loss and stress. *Frontiers in behavioral neuroscience* **7**, 224 (2013).

38. Tatsuki F*, et al.* Involvement of Ca(2+)-Dependent Hyperpolarization in Sleep Duration in Mammals. *Neuron* **90**, 70-85 (2016).

39. Sawamura N*, et al.* Nuclear DISC1 regulates CRE-mediated gene transcription and sleep homeostasis in the fruit fly. *Molecular psychiatry* **13**, 1138-1148, 1069 (2008).

40. Dauvilliers Y, Tafti M, Landolt HP. Catechol-O-methyltransferase, dopamine, and sleep-wake regulation. *Sleep medicine reviews* **22**, 47-53 (2015).

41. Naidoo N*, et al.* Role of Homer proteins in the maintenance of sleep-wake states. *PloS one* **7**, e35174 (2012).

42. Mauceri D, Freitag HE, Oliveira AM, Bengtson CP, Bading H. Nuclear calcium-VEGFD signaling controls maintenance of dendrite arborization necessary for memory formation. *Neuron* **71**, 117-130 (2011).

43. Rosch H, Schweigreiter R, Bonhoeffer T, Barde YA, Korte M. The neurotrophin receptor p75NTR modulates long-term depression and regulates the expression of AMPA receptor subunits in the hippocampus. *Proceedings of the National Academy of Sciences of the United States of America* **102**, 7362-7367 (2005).

44. Tam SK, Pritchett D, Brown LA, Foster RG, Bannerman DM, Peirson SN. Sleep and circadian rhythm disruption and recognition memory in schizophrenia. *Methods in enzymology* **552**, 325-349 (2015).

45. Hooper C, Killick R, Lovestone S. The GSK3 hypothesis of Alzheimer's disease. *Journal of neurochemistry* **104**, 1433-1439 (2008).

46. Barco A, Alarcon JM, Kandel ER. Expression of constitutively active CREB protein facilitates the late phase of long-term potentiation by enhancing synaptic capture. *Cell* **108**, 689-703 (2002).

47. Ying Z, Misra V, Verge VM. Sensing nerve injury at the axonal ER: activated Luman/CREB3 serves as a novel axonally synthesized retrograde regeneration signal. *Proceedings of the National Academy of Sciences of the United States of America* **111**, 16142-16147 (2014).

48. Canettieri G*, et al.* The coactivator CRTC1 promotes cell proliferation and transformation via AP-1. *Proceedings of the National Academy of Sciences of the United States of America* **106**, 1445-1450 (2009).

49. Fuchsova B, Alvarez Julia A, Rizavi HS, Frasch AC, Pandey GN. Altered expression of neuroplasticity-related genes in the brain of depressed suicides. *Neuroscience* **299**, 1-17 (2015).

50. Arai M*, et al.* Association of neural cell adhesion molecule 1 gene polymorphisms with bipolar affective disorder in Japanese individuals. *Biological psychiatry* **55**, 804-810 (2004).

51. Sheng L, Leshchyns'ka I, Sytnyk V. Neural cell adhesion molecule 2 promotes the formation of filopodia and neurite branching by inducing submembrane increases in Ca2+ levels. *The Journal of neuroscience : the official journal of the Society for Neuroscience* **35**, 1739-1752 (2015).

52. Noh KM*, et al.* Repressor element-1 silencing transcription factor (REST)-dependent epigenetic remodeling is critical to ischemia-induced neuronal death. *Proceedings of the National Academy of Sciences of the United States of America* **109**, E962-971 (2012).

53. Lee SE*, et al.* RGS14 is a natural suppressor of both synaptic plasticity in CA2 neurons and hippocampal-based learning and memory. *Proceedings of the National Academy of Sciences of the United States of America* **107**, 16994-16998 (2010).

54. Wei P, Blundon JA, Rong Y, Zakharenko SS, Morgan JI. Impaired locomotor learning and altered cerebellar synaptic plasticity in pep-19/PCP4-null mice. *Molecular and cellular biology* **31**, 2838-2844 (2011).

55. Ramakers GJ*, et al.* Dysregulation of Rho GTPases in the alphaPix/Arhgef6 mouse model of X-linked intellectual disability is paralleled by impaired structural and synaptic plasticity and cognitive deficits. *Human molecular genetics* **21**, 268-286 (2012).

56. Nakajima S*, et al.* The potential role of dopamine D(3) receptor neurotransmission in cognition. *European neuropsychopharmacology : the journal of the European College of Neuropsychopharmacology* **23**, 799-813 (2013).

57. Akiyama H*, et al.* Expression of BRI, the normal precursor of the amyloid protein of familial British dementia, in human brain. *Acta neuropathologica* **107**, 53-58 (2004).

58. Hayer SN, Bading H. Nuclear calcium signaling induces expression of the synaptic organizers Lrrtm1 and Lrrtm2. *The Journal of biological chemistry* **290**, 5523-5532 (2015).

59. Prediger RD*, et al.* Mice with genetic deletion of the heparin-binding growth factor midkine exhibit early preclinical features of Parkinson's disease. *Journal of neural transmission* **118**, 1215-1225 (2011).

60. Haberman RP, Lee HJ, Colantuoni C, Koh MT, Gallagher M. Rapid encoding of new information alters the profile of plasticity-related mRNA transcripts in the hippocampal CA3 region. *Proceedings of the National Academy of Sciences of the United States of America* **105**, 10601-10606 (2008).

61. Proenca CC, Gao KP, Shmelkov SV, Rafii S, Lee FS. Slitrks as emerging candidate genes involved in neuropsychiatric disorders. *Trends in neurosciences* **34**, 143-153 (2011).

62. Hou ZH, Yu X. Activity-regulated somatostatin expression reduces dendritic spine density and lowers excitatory synaptic transmission via postsynaptic somatostatin receptor 4. *The Journal of biological chemistry* **288**, 2501-2509 (2013).

63. Beesley PW, Herrera-Molina R, Smalla KH, Seidenbecher C. The Neuroplastin adhesion molecules: key regulators of neuronal plasticity and synaptic function. *Journal of neurochemistry* **131**, 268-283 (2014).

64. Alfonso J, Frick LR, Silberman DM, Palumbo ML, Genaro AM, Frasch AC. Regulation of hippocampal gene expression is conserved in two species subjected to different stressors and antidepressant treatments. *Biological psychiatry* **59**, 244-251 (2006).

65. Nonaka M, Kim R, Sharry S, Matsushima A, Takemoto-Kimura S, Bito H. Towards a better understanding of cognitive behaviors regulated by gene expression downstream of activity-dependent transcription factors. *Neurobiology of learning and memory* **115**, 21-29 (2014).

66. Chen WG*, et al.* Upstream stimulatory factors are mediators of Ca2+-responsive transcription in neurons. *The Journal of neuroscience : the official journal of the Society for Neuroscience* **23**, 2572-2581 (2003).

67. Ryan SM, O'Keeffe GW, O'Connor C, Keeshan K, Nolan YM. Negative regulation of TLX by IL-1beta correlates with an inhibition of adult hippocampal neural precursor cell proliferation. *Brain, behavior, and immunity* **33**, 7-13 (2013).

68. Quadrato G, Di Giovanni S. Waking up the sleepers: shared transcriptional pathways in axonal regeneration and neurogenesis. *Cellular and molecular life sciences : CMLS* **70**, 993-1007 (2013).

69. Meyer MA. Highly Expressed Genes within Hippocampal Sector CA1: Implications for the Physiology of Memory. *Neurology international* **6**, 5388 (2014).

70. Van Gool D, Carmeliet G, Triau E, Cassiman JJ, Dom R. Appearance of localized immunoreactivity for the alpha 4 integrin subunit and for fibronectin in brains from Alzheimer's, Lewy body dementia patients and aged controls. *Neuroscience letters* **170**, 71-73 (1994).

71. Lee CT, Ma YL, Lee EH. Serum- and glucocorticoid-inducible kinase1 enhances contextual fear memory formation through down-regulation of the expression of Hes5. *Journal of neurochemistry* **100**, 1531-1542 (2007).

72. Sultana R, Butterfield DA. Redox proteomics studies of in vivo amyloid beta-peptide animal models of Alzheimer's disease: Insight into the role of oxidative stress. *Proteomics Clinical applications* **2**, 685-696 (2008).

73. Elsir T, Smits A, Lindstrom MS, Nister M. Transcription factor PROX1: its role in development and cancer. *Cancer metastasis reviews* **31**, 793-805 (2012).

74. Chen M, Do H. Wnt Signaling in Neurogenesis during Aging and Physical Activity. *Brain sciences* **2**, 745-768 (2012).

75. Yang N, Wang Y, Hui L, Li X, Jiang X. Silencing SOX2 Expression by RNA Interference Inhibits Proliferation, Invasion and Metastasis, and Induces Apoptosis through MAP4K4/JNK Signaling Pathway in Human Laryngeal Cancer TU212 Cells. *The journal of histochemistry and cytochemistry : official journal of the Histochemistry Society* **63**, 721-733 (2015).

76. Takahashi K*, et al.* Induction of pluripotent stem cells from adult human fibroblasts by defined factors. *Cell* **131**, 861-872 (2007).

77. Prakash N, Wurst W. Genetic networks controlling the development of midbrain dopaminergic neurons. *The Journal of physiology* **575**, 403-410 (2006).

78. Deng H*, et al.* Examination of the MSX1 gene in patients with Parkinson's disease. *Acta neurologica Scandinavica* **120**, 442-444 (2009).

79. Holz A, Schaeren-Wiemers N, Schaefer C, Pott U, Colello RJ, Schwab ME. Molecular and developmental characterization of novel cDNAs of the myelin-associated/oligodendrocytic basic protein. *The Journal of neuroscience : the official journal of the Society for Neuroscience* **16**, 467-477 (1996).

80. Awsare NS, Martin TA, Haynes MD, Matthews PN, Jiang WG. Claudin-11 decreases the invasiveness of bladder cancer cells. *Oncology reports* **25**, 1503-1509 (2011).

81. Bronstein JM, Tiwari-Woodruff S, Buznikov AG, Stevens DB. Involvement of OSP/claudin-11 in oligodendrocyte membrane interactions: role in biology and disease. *Journal of neuroscience research* **59**, 706-711 (2000).

82. Narayan S, Kass KE, Thomas EA. Chronic haloperidol treatment results in a decrease in the expression of myelin/oligodendrocyte-related genes in the mouse brain. *Journal of neuroscience research* **85**, 757-765 (2007).

83. Park K*, et al.* ER stress stimulates production of the key antimicrobial peptide, cathelicidin, by forming a previously unidentified intracellular S1P signaling complex. *Proceedings of the National Academy of Sciences of the United States of America* **113**, E1334-1342 (2016).

84. Pound LD*, et al.* Cathelicidin Antimicrobial Peptide: A Novel Regulator of Islet Function, Islet Regeneration, and Selected Gut Bacteria. *Diabetes* **64**, 4135-4147 (2015).

85. Koczulla R*, et al.* An angiogenic role for the human peptide antibiotic LL-37/hCAP-18. *The Journal of clinical investigation* **111**, 1665-1672 (2003).

86. Nagaoka I, Tamura H, Hirata M. An antimicrobial cathelicidin peptide, human CAP18/LL-37, suppresses neutrophil apoptosis via the activation of formyl-peptide receptor-like 1 and P2X7. *Journal of immunology* **176**, 3044-3052 (2006).

87. Bhandari V*, et al.* Hyperoxia causes angiopoietin 2-mediated acute lung injury and necrotic cell death. *Nature medicine* **12**, 1286-1293 (2006).

88. Liu A, Jain N, Vyas A, Lim LW. Ventromedial prefrontal cortex stimulation enhances memory and hippocampal neurogenesis in the middle-aged rats. *eLife* **4**, (2015).

89. Mehedint MG, Craciunescu CN, Zeisel SH. Maternal dietary choline deficiency alters angiogenesis in fetal mouse hippocampus. *Proceedings of the National Academy of Sciences of the United States of America* **107**, 12834-12839 (2010).

90. Lim DC*, et al.* Different Cyclical Intermittent Hypoxia Severities have Different Effects on Hippocampal Microvasculature. *Journal of applied physiology*, jap 01040 02015 (2016).

91. Squillario M, Barla A. A computational procedure for functional characterization of potential marker genes from molecular data: Alzheimer's as a case study. *BMC medical genomics* **4**, 55 (2011).
